# Effectiveness of interpersonal psychotherapy in comparison to other psychological and pharmacological interventions for reducing depressive symptoms in women diagnosed with postpartum depression in low‐ and middle‐income countries: A systematic review

**DOI:** 10.1002/cl2.1399

**Published:** 2024-04-21

**Authors:** Harmeet Kaur Kang, Bandana Bisht, Manmeet Kaur, Obrey Alexis, Aaron Worsley, Denny John

**Affiliations:** ^1^ Chitkara School of Health Sciences Chitkara University Punjab India; ^2^ Department of Psychiatry Government Medical College & Hospital Chandigarh India; ^3^ Shaheed Kartar Singh Sarabha College of Nursing Sarabha Ludhiana India; ^4^ Oxford Brookes University Swindon UK; ^5^ Faculty of Life and Allied Health Sciences M S Ramaiah University of Applied Sciences Bangalore Karnataka India

## Abstract

**Background:**

Postpartum depression (PPD) is a condition that can affect any woman regardless of ethnicity, age, party, marital status, income, and type of delivery. This condition is highly prevalent worldwide. PPD, if not treated timely, can affect the maternal‐child bond and can have a detrimental impact on the future cognitive, emotional, and behavioral development of the child. Interpersonal psychotherapy (IPT) has been reported as an effective treatment of PPD in previous studies as this focuses on relationship and social support issues. Previous reviews conducted in developed nations have reported the superior efficacy of IPT in comparison to other treatment options. There is no systematic review conducted in low to middle‐income countries on the efficacy of IPT on PPD. Therefore it was necessary to undertake a systematic review to assess the effectiveness of IPT in reducing the depression among postpartum women in low and middle‐income countries (LMICs).

**Objectives:**

The main aim of this systematic review was to assess the effectiveness of IPT alone or in conjunction with pharmacological therapy and/or other psychological and psychosocial interventions, in reducing depressive symptoms among women diagnosed with PPD residing in LMICs.

**Search Methods:**

The systematic search encompassed several prominent databases and grey literature. Furthermore, experts specializing in the field of IPT were consulted to identify any relevant studies conducted in LMICs that fulfilled the predetermined eligibility criteria. The most recent search update was performed in July 2022.

**Selection Criteria:**

The PICOS criteria were meticulously defined for this review as described. Participants: Postpartum women diagnosed with PPD in LMICs were included. Intervention: IPT either as a standalone treatment or in conjunction with pharmacological therapy was included. Comparison: any form of psychological therapy or pharmacological therapy, whether administered individually or in combination, was considered for comparison. Study designs: experimental and quasi‐experimental, factorial designs, and quantitative components (experimental, quasi‐experimental, factorial designs) of mixed methods designs were eligible to be included. Studies with single‐group study designs and qualitative studies were excluded from the review.

**Data Collection and Analysis:**

Two reviewers from our team conducted a rigorous screening process to determine the eligibility of articles for inclusion. This involved an initial evaluation of titles and abstracts, followed by a comprehensive assessment of the full text of selected articles. In instances where discrepancies arose between the two reviewers, resolution was achieved through discussion or consultation with a third author to establish a consensus. Following the screening process, two team members independently extracted pertinent information and data from the studies that met the inclusion criteria. The treatment effect of the intervention, in comparison to the control group, was subsequently analyzed utilizing the fixed effects model taking into account the small number of studies.

**Main Results:**

A total of 17,588 studies were identified from various databases, and 6493 duplicate studies were removed. Subsequently, 9380 studies underwent independent title and abstract screening resulting in the exclusion of 9040 studies. 345 full texts were thoroughly assessed leading to the exclusion of 341 studies, finally including 4 studies for review. The four included trials were randomized trials and comprised a total sample size of 188 women diagnosed with PPD residing in LMICs. Among these studies, three compared IPT with usual treatment, while one study compared IPT with antidepressant medications (ADMs). In terms of the providers of IPT, in one study, IPT was administered by nurses, while psychologists delivered IPT in another study. In one study, community health workers were responsible for providing IPT. However, in one study, information regarding the specific providers of IPT was not available or reported. The primary outcome measure reported in all four studies was depression, assessed using the Edinburgh Postnatal Depression Scale (EPDS). The geographical distribution of the studies included; one conducted in Zambia, one in Kenya, one in Pakistan, and one in Iran. Out of the four studies, three were included in the meta‐analysis, as missing data from one study could not be obtained. Based on the overall treatment effect, it was found that depression scores decreased significantly more in the IPT group compared to other interventions (usual treatment or ADMs) (standardized mean difference [SMD] −0.62, 95% confidence interval [CI] (−1.01, −0.23), *Z* = 3.13 (*p* = 0.002), *χ* ^2^ = 49.49; df = 2; *p* < 0.00001; *I* ^2^ = 96%; 3 studies, *n* = 136). Out of the three studies, two studies compared the effectiveness of IPT in reducing depression scores specifically when compared to the usual treatment, and in both studies, depression scores were reduced significantly in the IPT group as compared to the usual treatment group. Only one study directly compared the effectiveness of IPT with ADM, reporting that IPT was more effective than ADM in reducing depression scores among postpartum women. Regarding adverse outcomes, only one study reported suicidal ideation with one participant in the IPT group and two in the ADM group (RR 0.50, 95% CI (0.05, 5.30), *p* = 0.56, *n* = 78). The same study reported seven participants in the ADM group had adverse drug reactions as compared to none in the IPT group (RR 15.0, 95% CI (0.89, 254), *p* = 0.06, *n* = 78).

**Authors' Conclusions:**

Our comprehensive search yielded a limited number of four studies conducted in such settings. Despite the scarcity of available evidence, the findings collectively suggest that IPT is indeed an effective treatment for reducing PPD when compared to usual treatment and pharmacological therapy. However given the low certainty of evidence, there is a need for further research in the form of well‐designed randomized controlled trials with larger sample sizes and a reduced risk of bias. Such studies would greatly contribute to enhancing the strength and reliability of the evidence base regarding the effectiveness of IPT in the context of PPD in LMICs. The knowledge generated from future research endeavors would be highly valuable in guiding the development of more affordable and cost‐effective treatment approaches for PPD in resource‐limited settings.

## 1 PLAIN LANGUAGE SUMMARY

### 1.1 Postpartum depression (PPD)

Depression is a common mental health issue that many women experience during their childbearing years. It can manifest as persistent feelings of sadness and emptiness that last for more than 2 weeks. When this type of depression occurs after childbirth, it is called PPD. This condition can have a significant impact on a mother's overall well‐being and quality of life.

### 1.2 What is this review about?

PPD is a common issue that affects women after giving birth. There are various reasons why women may experience this, such as unplanned pregnancy, lack of support from family, or having a low income. Many women who have PPD find that psychological therapy is their preferred choice of treatment instead of taking medication.

Interpersonal psychotherapy (IPT) is a type of therapy that has been shown to be effective in treating different mental health disorders, including PPD. In this review, we aim to investigate whether IPT can help reduce depression among women who have recently given birth and live in low and middle‐income countries (LMICs). By examining the available evidence, we hope to gain insights into the effectiveness of this therapy for postpartum women in these specific settings.

### 1.3 What is the aim of this study?

We aimed to find out whether IPT alone or in combination with pharmacological therapy is effective in reducing depression symptoms in comparison to other psychosocial or pharmacological treatments in postpartum women residing in LMICs.

### 1.4 What studies are included?

Seven databases including Cochrane Central Registry of Controlled Trials, PUBMED, EMBASE, PsycINFO, ERIC(ProQuest), CINAHL, and Web of Science. These databases were searched to identify relevant studies conducted and published between January 1970 to July 2022. A total of 9380 titles and abstracts were screened followed by 345 full‐text screening and finally, only 4 studies passed the quality measures and were deemed appropriate for the systematic review. The meta‐analysis incorporated only three out of four studies, as one study could not be included due to insufficient data availability. Among the four studies, two were carried out in African countries, one in Iran, and one in Pakistan.

### 1.5 What are the main findings of the review?

The four included studies had a total sample of 188 women suffering from PPD. The IPT (ranging between 8 and 12 sessions) was given to postpartum mothers by psychologists, nurses, and community health workers and was found to be effective in a greater reduction in PPD scores in comparison to usual treatment and antidepressant therapy.

### 1.6 What do the findings of this review mean?

Based on our review findings, IPT could be helpful in reducing depression among postpartum women in LMICs. This finding contributes to our understanding of affordable and cost‐effective treatment options for PPD in low‐resource settings.

To further enhance our knowledge in this area, future research can focus on identifying the specific components of IPT that are most effective, the best ways to deliver the therapy, the optimal number of sessions needed, and guidelines for making the therapy more feasible, acceptable, and easily implemented for treating PPD in LMICs.

## 2 SUMMARY OF FINDINGS

### 2.1 Summary of findings 1

Summary of findings

**Table:** 

Interpersonal Psychotherapy compared to Other interventions (Usual Treatment or Antidepressant Medications for Postpartum women with Depression in low‐middle income countries						
**Patient or population:** Postpartum women with depression residing in low‐middle‐income countries						
**Setting:** Any setting in a hospital/community health center in low‐middle‐income countries						
**Intervention:** Interpersonal Psychotherapy						
**Comparison:** Other interventions (Usual Treatment or Antidepressant Medications)						

	Anticipated absolute effects* (95% CI)					
Outcomes	Risk with Other interventions (Usual Treatment or Antidepressant Medications	Risk with Interpersonal Psychotherapy	Relative effect (95% CI)	No. of participants (studies)	Certainty of the evidence (GRADE)	Comments
Depression Scores (Depression) assessed with: Edinburgh Postnatal Depression Questionnaire (EPDS)	‐	SMD **2.7** (5.26 lower to 0.14 lower)	‐	188 (3 RCTs)	⨁◯◯◯ Very low^1,2^	Depression scores reduced significantly in the interpersonal psychotherapy group as compared to other groups (usual/antidepressant medication)
Maternal Adjustment (Maternal Adjustment)	Marriage adaptation improved in the interpersonal psychotherapy group as compared to the usual treatment group.			34 (1 RCT)	⨁◯◯◯ Very low^1^	
Suicidal Ideation (Suicidal Ideation)	**Study population**		**RR 0.50** (0.05 to 5.30)	78 (1 RCT)	⨁⨁⨁◯ Moderate^3^	
	26 per 1,000	**13 per 1000** (1 to 136)				
	**Low**					
	26 per 1000	**13 per 1000** (1 to 138)				
Adverse Drug Reaction (Adverse Drug Reaction)	**Study population**		not estimable	78 (1 RCT)	⨁⨁⨁◯ Moderate^3^	
	179 per 1000	**0 per 1000** (0 to 0)				
	**High**					
	179 per 1000	**0 per 1000** (0 to 0)				

*The risk in the intervention group (and its 95% confidence interval) is based on the assumed risk in the comparison group and the **relative effect** of the intervention (and its 95% CI).

**CI:** confidence interval; **RR:** risk ratio; **SMD:** standardized mean difference

**GRADE Working Group grades of evidence**

**High certainty:** we are very confident that the true effect lies close to that of the estimate of the effect.

**Moderate certainty:** we are moderately confident in the effect estimate: the true effect is likely to be close to the estimate of the effect, but there is a possibility that it is substantially different.

**Low certainty:** our confidence in the effect estimate is limited: the true effect may be substantially different from the estimate of the effect.

**Very low certainty:** we have very little confidence in the effect estimate: the true effect is likely to be substantially different from the estimate of effect.

References

1. Mahnaz Hajiheidari, Marzieh Sharifi, Fariborz Khorvash. The Effect of interpersonal psychotherapy on marriage adaptive and postpartum depression in Isfahan. *International Journal of Preventive Medicine*; 2013.

2. Yator Obadia, John‐Stewart Grace,Khasakhala Lincoln, Kumar Manasi. Preliminary Effectiveness of Group Interpersonal Psychotherapy for Young Kenyan Mothers With HIV and Depression: A Pilot Trial. *The American Journal of Psychotherapy*; 2021.

3. M. Bridget Spelke, Ravi Paul, Bryan S. Blette, Samantha Meltzer‐Brody, Crystal E. Schiller, J. M. Ncheka, Margaret P. Kasar, Joan T. Price, Jeffrey S. A. Stringer, and Elizabeth M. Stringer. Interpersonal therapy versus antidepressant medication for treatment of PPD and anxiety among women with HIV in Zambia: a randomized feasibility trial. *Journal of the International AIDS Society*; 2022.

## 3 BACKGROUND

Depression is the most common mental health condition affecting most perinatal women and mothers (Hanlon, 2013a). It is estimated that 15%–85% of women experience postpartum blues within 10 days of giving birth which is considered as a subsequent risk factor for PPD (Hunsley, 2014). Available evidence suggests that one out of every seven women suffers from PPD US Preventive Services Task Force (USPSTF, 2019). In psychiatric nomenclature, PPD is defined as a major depressive disorder with postpartum onset. According to the Diagnostic Statistical Manual 5 (DSM 5) (American Psychiatric Association, 2013), PPD is defined as, a major depression episode during the period of pregnancy or within 4 weeks of delivery. Whereas, the International Classification of Diseases 10 (ICD 10) guidelines state that the onset of PPD is considered to be within 6 weeks post‐delivery (WHO, 2016). This condition is more prevalent in LMICs compared to high‐income countries (HICs) (Gajaria, 2018). Furthermore, PPD is known to affect mothers regardless of the fact that they may have had an easy or problematic pregnancy (Lipsey, 2001; Markowitz, 2004). It has been reported that approximately 19% of mothers from LMICs experience significant depressive symptoms during the first 3 months after giving birth. PPD in LMICs is highly prevalent affecting every one out of five women (Gao, 2010a; Gelaye, 2016a). The prevalence rate of PPD in LMICs is 31.8% which is comparably higher than in developed countries which is, 21.5%. Also, the prevalence of PPD in LMICs among rural women is higher than the rural women of developed countries (Fisher, 2012). Women experiencing PPD may exhibit symptoms such as depressed mood, anxiety, feelings of guilt or inadequacy, inability to cope, loss of control, intrusive presence of compulsive thoughts, irrational fears, and despair (American Psychiatric Association, 2013). Moreover, PPD can predispose to chronic or recurrent depression, which may affect the mother‐infant relationship and the child's growth and development (Miniati, 2014). Untreated depression in mothers is associated with public health and social consequences for mothers and children. In South Asia, maternal depression was found to be an independent risk factor for infant malnutrition and diarrheal episodes (Hanlon, 2013a). Children of mothers with PPD have greater cognitive, behavioral, and interpersonal problems compared with children of non‐depressed mothers (Slomian, 2019a). Pregnancy is considered a sensitive period where various bio‐psychosocial variants influence the psychology of a pregnant woman (Slomian, 2019a). The significant predictors causing PPD include biological factors. Studies demonstrated that younger women were more likely to experience PPD (Ghaedrahmati, 2017a). Additionally, women with glucose metabolism disorders were at risk of developing PPD (Huang, 2015a). Moreover, changes in role transitions and interpersonal factors (e.g., partnership satisfaction, social support) during or after pregnancy are contributory factors to developing PPD (Martini, 2015). In addition, several social factors leading to PPD are unmarried mothers, unplanned pregnancy, lack of support from partners, parents, or relatives during the postpartum period and impaired interpersonal relationships with the spouse have been shown to contribute towards PPD among women in both LMICs and HICs. Postpartum mothers with lower socioeconomic status and primigravida frequently encounter a higher prevalence of PPD and may occasionally consider seeking IPT. Originally designed for individuals with depression, IPT has undergone adaptations to cater to various groups and can be provided through telephone sessions or extended to couples (Markowitz, 2004). IPT reduces symptoms of depression and improves relationship quality, social adjustments, and social support, however, there is insufficient evidence on the efficacy of different adaptations of IPT used for perinatal women (Sockol, 2018; Bright, 2020). High‐middle‐income people residing in LMICs usually do not have access to health services, good nutrition, or a clean environment (Bener, 2012). There are no viable guidelines in LMICs for treating PPD. Hence, WHO suggests integrating mental health services with primary care so as to reduce the treatment gap for LMICs. The usual treatment for PPD involves psychotherapy and antidepressants. Due to concerns regarding infant exposure to antidepressants, the preferred choice of treatment for mothers is psychotherapy (O'Hara, 2000; Stuart, 2013). IPT is considered the most relevant to PPD as IPT targets the specific interpersonal problems experienced by women in the postpartum period. The time of receiving IPT for PPD range from 0.5 weeks to 96 weeks post‐partum (Bright, 2020). IPT is based on the fact that interpersonal problems are encountered during the period of pregnancy along with various hormonal changes which result in possible symptoms of depression (Deeks, 2011). Treatment modalities delivered by mental health care professionals in low and high‐middle‐income countries for mothers with PPD can help achieve positivity in terms of better therapeutic outcomes along with psychosocial improvement (Markowitz, Markowitz 2004, Miniati 2014; Yuksel, 2015). Many studies have demonstrated the efficacy of IPT in depression over other psychological treatments (Beeber, Beeber 2013, Bener 2012a; Bolton, 2003; Markowitz, Markowitz 2004, Miniati 2014; Posmontier, 2016). Various organizations such as the American Psychiatric Association and the National Institute for Health and Clinical Excellence: Guidance (NICE, 2014), have recognized IPT as an efficacious psychological therapy. Despite the growing number of empirical studies on PPD, there is a lack of robust systematic evidence on the efficacy of IPT to treat PPD among LMICs. Therefore, this systematic review will determine the usefulness of IPT to treat PPD among mothers in LMICs.

### 3.1 Description of the condition

Depression is more common among women than in men, especially during childbearing years (Dennis, 2020). Motherhood is a joyous period for many women, but it also brings immense emotional, biological, social, and financial changes (Torres, 2020). It can trigger a variety of emotions varying from elation to sadness, or excitement to apprehension, hence making most women vulnerable to poor mental health. Most women may experience baby blues comprising sadness and emptiness which usually disappear in 3–5 days. If the symptoms persist for longer than 2 weeks, it could be PPD (Markowitz, 2014, 2012; American Psychiatric Association, 2013; Pearlstein, 2009). PPD is a common health concern for many women, yet it remains undiagnosed, affecting maternal and child health. The symptoms of PPD usually are visible within the first 4 weeks after birth and if not resolved on time, may intensify in the next 6 months (Bener, 2012a). Depression is one of the most common forms of maternal morbidity. It can affect any woman regardless of her age, type of delivery, marital status, income, ethnicity, or educational status (Dennis, 2020). Untreated post‐partum depression may persist until their children start going to school (Bright, 2020). PPD has a salient effect on mother‐infant relationships. It also affects a child's emotional, cognitive, and behavioral development (Gaillard, 2014). The prevalence of PPD among mothers living in urban areas is 20.4% and in rural areas, it is 15.8% (Gao, 2010a). The 12‐month prevalence of PPD in HICs is 5.5% whereas in LMICs it is 5.9% (Hajiheidari, 2013). PPD is said to be managed with the help of psychotherapy, psychopharmacology, or sometimes a combination of both.

Despite the growing number of empirical studies on PPD (Bener, 2012a), there is a lack of robust systematic evidence on the efficacy of IPT to treat PPD among LMICs. Therefore, the present systematic review will determine the usefulness of IPT to treat PPD among women living in LMICs.

### 3.2 Description of the intervention

IPT has originally been developed by Dr. Gerald Klerman, Myra Weissman, and their colleagues (Markowitz, Markowitz 2004, Miniati 2014). Earlier, it was used for the treatment of depression but, subsequently, it was adapted for the use of other mental disorders such as bipolar disorder, anxiety disorders, substance misuse disorders, and eating disorders (Alhusen, 2016). It is one of the therapies recommended for major depression by the American Psychiatric Association and Guidelines for Primary Care Physicians (Edmond, 2017). American Academy of Pediatrics has recommended IPT as a treatment choice for adolescents with depression. In 2016, the WHO recommended it as the first‐line treatment for post‐partum depression (Stangier, 2011b).

Life events such as the death of a significant person (grief/loss), antagonistic relationships (role disputes), disruptions in life (role transitions), and isolation or lack of social support (interpersonal deficit) make the individual vulnerable to falling into depression (Ravitz, 2019). IPT explores the patterns of interpersonal relationships and sees how these patterns cause difficulty in relationships and the patient's response to such events. Hence the treatment goals revolve around teaching different ways of interaction, thus changing the affective experience of loss, life transitions, role disputes, and social isolation in an individual.

#### 3.2.1 Duration of the course

The standard protocol for IPT typically consists of 12–16 weekly sessions. While IPT is considered a time‐limited therapy, its duration can be adjusted to accommodate various situations and conditions, offering flexibility to clients. Alternatively, a condensed version known as Brief IPT is available, consisting of only 8 sessions. This abbreviated format was developed due to limited evidence supporting the notion that longer treatment durations lead to superior outcomes (Webster, 2011). Brief IPT enables clients to derive the benefits of IPT within a shorter timeframe, particularly when faced with constraints or limitations. The therapy can be administered in multiple modalities, as described below.

#### 3.2.2 Types of IPT

IPT can be delivered in various formats such as:

1. Individual IPT
2. Group IPT
3. IPT for couples or Conjoint Marital Interpersonal therapy (CM‐IPT)
4. Telephone‐administered IPT

#### 3.2.3 Settings for IPT

1. Healthcare settings
2. Community settings

IPT can be delivered by individuals with diverse professional backgrounds who have received appropriate training in IPT methodology. This includes not only specialists in mental health such as psychiatrists, psychologists, social workers, and mental health nurses, but also non‐specialists who have undergone adequate training. Non‐specialists may include community health workers, general physicians, nurses, and individuals holding degrees in social or behavioral sciences. While IPT is most commonly administered by mental health professionals, the expanding accessibility of IPT training allows a broader range of professionals to effectively provide this therapy.

Below are the four problem areas where IPT can be offered to mothers with PPD:

1. *Grief* – Grief is the loss (death) of someone significant to the client. For example, death of spouse, child, parent, close friend or relative (sometimes a pet also). In usual course, a normal grief period lasts for few months. In some cases, the person may have complicated or difficult relationship with the person who has died, resulting in the prolonged period of distress. Individual may have symptoms of depression (e.g., suicidal ideation, worthlessness, motor retardation, slow speech, loss of interest).
2. *Disputes* – Disagreement with someone in a person's life which means that the person in disagreement might be fighting openly or maybe avoiding conflict, however significant. The goal for this problem area is to help the person figure out what they and the other party expect culminating in developing effective communication skills and practicing those skills alongside mobilizing people who can help bring about a resolution of the conflict.
3. *Life Changes/Role transition* – It includes changes in the person's role or expectation of changes, either positive or negative, which in turn is having an effect on the relationship. For example, marriage, moving to a new house, new job, retirement, childbirth, separation or rejection by partner, serious illness, or financial crisis. The goal is to examine the positive side of the change, to learn the skills necessary to manage the change, and, to find support to manage the change.
4. *Loneliness/Social isolation* – The client will verbalize the chronic feelings of loneliness, boredom, and emotional distance from others. There will be a history of poor relationships with family, friends, or relatives. Though the problem area is longstanding, it worsens when any of the other three problem areas arise in the client's life. The goal in this problem area would be to find out what contributes to their loneliness and to learn new ways to begin and maintain relationships (American Psychiatric Association, 2013).

#### 3.2.4 Phases of IPT

There are two phases of IPT – The intensive phase and the maintenance phase.

##### The intensive phase

It has three parts:

1. Initial Phase
2. Middle Phase
3. Termination Phase

**Table:** 

Phases	Initial Phase	Middle Phase	Termination Phase
Sessions	1–3	4–14	14/15–16

###### Initial phase

In the initial phase, the client and the therapist get to know each other and set a treatment plan. The therapist completes a psychosocial history and assesses the symptoms of distress, physical and psychological. The therapist assesses risk of suicide. Before initiating therapy, the therapist rules out bipolar disorder, psychosis, cognitive impairment, and serious substance abuse.

Once the therapist identifies the problem area, the time limit and frequency of sessions is explained to the client. The time limit for each session is usually 1 h. The session is conducted once a week.

###### Middle phase

This is the most active phase of therapeutic relationship. The therapist reviews the symptoms of depression every week on any of standardized scale and then it is seen in context of the identified problem area. The therapist helps the client to link the onset of symptoms with the problem area and help them to work on selected strategies.

###### Termination phase

The termination phase occurs during the last 2–3 sessions. Here, the therapist points at the achievement of the client during the middle sessions by learning new coping strategies and new communication technique which gives him/her the sense of competence and confidence. Therapist also summarizes the nature of depression, chances of recurrence and importance of monitoring their symptoms.

##### The maintenance phase

Usually, the person gets treated in the initial phase only but in some cases maintenance phase may be required. For example, if there is

- Partial improvement
- History of recurrent depression
- Unwillingness to take medicine
- Renegotiation on the length of the treatment
- Renegotiation on the focus of problem area

### 3.3 How the intervention might work

**Figure 1:**
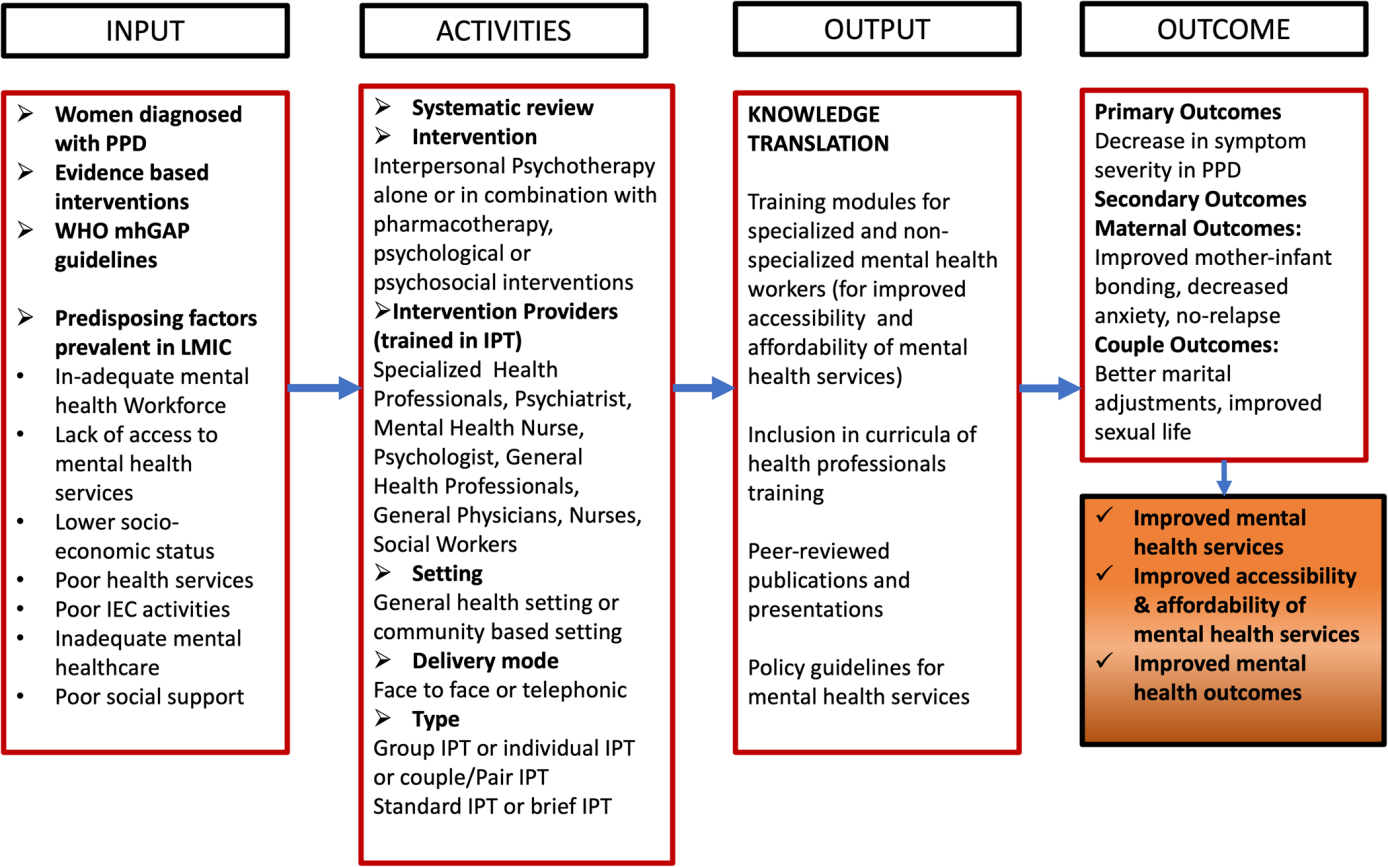
Logic Model concerning the effectiveness of interpersonal psychotherapy to improve postpartum depression outcomes and improved mental health services in LMICs.

Psychotherapy is a better treatment than pharmacotherapy for depression as there are fewer relapses and lesser premature termination of treatment (Hunsley, 2014). Emerging evidence suggests better treatment adherence and decreased suicides if psychotherapy is added along with medication for the treatment of depression (Hunsley, 2014). Comparison of various types of psychotherapies like cognitive behavior therapy (CBT), cognitive therapy, behavior therapy, play therapy, psychodynamic therapy, family therapy, problem‐solving therapy, supportive therapy, and IPT has been examined in a number of clinical trials (Stamou, 2018). CBT and IPT are more efficacious in treating depression as compared to other therapies (Cuijpers, 2016). Though IPT and CBT have equal or nearly equal effectiveness (Cuijpers, 2016; Power, 2012), however, IPT has some added advantages over CBT (WHO, 2019). IPT can be given by non‐specialized trained persons (Van Ginneken, 2013; WHO, 2016). Unlike other therapies, the flexible approach of IPT increases its scope in terms of accessibility and affordability to a larger population. That is why WHO has recommended IPT as the first‐line treatment for depression for pregnant and breastfeeding mothers with moderate‐severe depressive disorder (WHO, 2016b) (Figure 1).

The logic model has been adapted from Ludwig's systems model (Von Bertalanffy, 1971), where different factors influencing the outcome of treatment strategy on PPD are identified in LMICs. Potential influencing factors such as existing literature and evidence, WHO guidelines, and predisposing factors in LMICs for the occurrence of PPD are presented. The intervention for the treatment of PPD could be IPT alone or IPT with pharmacotherapy or IPT in combination with other psychological/psychosocial interventions in general health settings or community‐based settings (Bright, 2020). The mode of delivery could be a face‐to‐face or telephonic conversation. IPT can be provided by specialized health workers such as psychiatrists, psychologists, or mental health nurses, or non‐specialized general health workers such as general physicians, nurses, and social workers who are trained in IPT. IPT can be given in groups or individually. When 6–10 members are part of the therapy at one time, it is called Group‐IPT. The activities also include differentiation on the basis of duration of the therapy, that is,Standard IPT, which ranges between 12 and 16 weeks, and Brief IPT, that is, of 8 weeks. The primary outcome of giving IPT would be a decrease in symptom severity among the mothers suffering from PPD. The secondary outcomes can fall under maternal outcomes and couple outcomes. Maternal outcomes can be measured in the form of improved mother‐infant bonding, decreased anxiety of the mother, and no future relapse. IPT for couples, also termed as conjoint marital IPT can lead to better couple outcomes in relation to better marital adjustments and improved sexual life. Relationship disputes in postpartum period may result from unequal division of child care responsibilities and household work. Relationship distress arising because of inadequate partner support can be a risk factor for PPD. IPT for couples is attended by both partners together except one session in the initial phase of IPT which is supposed to be an individual session with the therapist (Carter, 2010). IPT has evolved as an effective treatment for various psychiatric disorders across various life spans, in different formats and for diverse clinical populations (Ravitz, 2019). IPT can lead to improved mental health outcomes. It impacts the community at large by providing accessible and affordable mental health treatment.

### 3.4 Why it is important to do this review

PPD is characterized by the onset within 4 weeks of delivery. As per American Psychiatric Association, the former is still under‐acknowledged and under‐researched mainly in LMICs (American Psychiatric Association, 2013). The primary aim of this present review is to focus on IPT especially in LMICs due to a lack of available evidence in this particular area. Furthermore, there is a lack of resources mainly in healthcare facilities, and a prevailing treatment gap, especially in LMICs (Basu, 2012). Previously available reviews have evaluated the effectiveness of psychological and psychosocial treatments based on PPD (Abdul, 2018). Existing reviews have closely focused on symptom reduction rather than comprehensive interventional modalities related to PPD (Stuart, 2012). No supporting literature has focused on the mode and duration of IPT along with a combination of other interventions. A systematic review by Miniati (2014) shows the effectiveness of IPT in relieving symptoms of PPD (Miniati, 2014). However, very few studies have reported the effectiveness of combining IPT with pharmacotherapy in reducing the severity of PPD. A systematic review was conducted specifically to examine the treatment outcomes based on IPT in reducing PPD Blackmore (2012). However, the review was limited in its applicability to LMICs.

Very few reviews have focused on the prevention of PPD (Dennis, 2009). As per the manpower census report by World Health Organization, mental health experts and health providers mainly in LMICs is comparatively very low compared to the developed territories (WHO, 2016). As per World Health Organization, IPT or CBT should be considered as a first‐line treatment for expecting and lactating mothers with depression. CBT can be delivered by any trained psychologist or clinical psychologist. General health professionals can not provide CBT. IPT can be delivered by mental health professionals or general health professionals after receiving appropriate training in IPT (WHO, 2016b). In LMICs, there are 0.9–14.7 mental health workers per 100,000 population, as compared to 62.2 per 100,000 in HICs (World, 2021). Due to this huge scarcity of professionals delivering mental health interventions (Bruckner, 2011), training general health workers in IPT can be useful. According to WHO's comprehensive mental health action plan year 2013–2020, IPT could be considered an accessible therapy in comparison to other psychotherapies as it can be further implemented in both general and community‐based healthcare settings (Fisher, 2012).

PPD affects one in every five women. Developed countries or HICs have low prevalence rates of PPD as compared to LMICs (Prom, 2022; Wang, 2021). A recent systematic review on “Mapping global prevalence of depression among post‐partum women” found that there is a significantly low number of research studies on PPD conducted in LMICs (Wang, 2021). The effect of the individual approach versus the integrated approach that combines psychosocial/psychological treatments with or without medications for the treatment of maternal depression is highly warranted (Gajaria, 2018a). There is a need to scale up the treatment of PPD in LMICs which also means that there is a need to understand the effectiveness of evidence‐based treatments such as IPT and its various adaptations in comparison to other routinely used treatments such as pharmacotherapy, and CBT to name but a few. The present systematic review will focus on the effectiveness of IPT alone or in combination with pharmacotherapy and/or other psychological or psychosocial interventions or other psychosocial intervention with pharmacotherapy for treating depressive symptoms. The review would be useful in generating insights into the role of IPT in treating depressive symptoms in LMICs.

## 4 OBJECTIVES

### 4.1 Primary objective

(a) What is the effectiveness of IPT alone or in combination with pharmacological therapy and/or other psychological and psychosocial interventions in reducing depressive symptoms in women diagnosed with PPD?

### 4.2 Secondary objectives

(b) What is the effectiveness of IPT according to the mode, that is, individual, group, conjoint and telephonic on reducing depressive symptoms in PPD?
(c) What is the effectiveness of standard IPT (12–16 sessions) versus brief IPT (8 sessions) on reducing depressive symptoms in PPD?

## 5 METHODS

### 5.1 Criteria for considering studies for this review

#### 5.1.1 Types of studies

The review is based on the protocol published (Kang, 2020). Randomized controlled trials (RCTs), feasibility trials, and quasi‐experimental designs were eligible for this study. Quasi‐experimental designs such as controlled before‐after design and difference in difference designs with homogenous experimental and comparison groups only were planned to be included in the review. We planned to include quantitative components of mixed methods research and factorial designs but found no studies. Study designs such as case reports, case series, reviews, and non‐original studies such as editorials, book reviews, commentaries, and letters to editors, were excluded. Qualitative research designs were also excluded.

#### 5.1.2 Types of participants

The specific population of interest was postpartum mothers in LMICs diagnosed with depression developed during pregnancy or within 6 weeks post‐delivery, identified either through clinical diagnosis (DSM‐IV, ICD‐10) or self‐reported measures, for example, Beck Depression Inventory (BDI), Edinburgh Postnatal Depression Scale (EPDS), Public Health Questionnaire (PHQ‐9), Hamilton Rating Scale for Depression (HRSD), Postpartum Depression Scale (PPDS), Major Depression Inventory (MDI), Inventory to Diagnose Depression (IDD), Center for Epidemiological Studies Depression Scale (CES‐D) and Zung Self‐rating depression scale (SDS).

Our review was international in scope and was applicable to LMICs only.

#### 5.1.3 Types of interventions

Interventions for the review included IPT alone or in combination with the pharmacological treatment given to women with PPD.

It was planned to divide the studies into categories to examine the different modes of delivering IPT such as Group‐IPT versus Individual IPT, Brief IPT/IPT‐B (8 weeks) versus IPT (12–16weeks), Telephonic IPT versus Standard IPT, and subgrouping into categories to examine the effect of IPT intervention delivered by a psychiatrist, psychologist, trained nurses/social workers/health visitors and non‐specialized trained volunteers from the community. But due to the small number of included studies, a subgroup/moderator could not be conducted.

#### 5.1.4 Types of outcome measures

The primary and secondary outcomes for the review were:

##### Primary outcomes

Depressive symptoms such as severity, symptom remission, recovery status, and relapse are measured through clinical diagnosis (DSM or ICD criteria) or self‐reported measures, for example, BDI, EPDS, PHQ‐9, HRSD, PPDS, MDI, IDD, CES‐D and Zung SDS.

##### Secondary outcomes

We planned to assess the secondary outcomes; maternal outcomes: mother‐infant bonding/attachment, anxiety, social support, postpartum adjustment, social adjustment, transition back to work; couple outcomes: marital adjustment, sexual interest, and adverse effects outcomes: suicide attempts, suicides, number of hospitalizations, duration of hospitalization, lost workdays, and so forth.

###### Duration of follow‐up

Any duration of follow‐up was planned to be included.

###### Types of settings

All general healthcare and community‐based settings were included. We also included the study settings where telephone‐based interventions were given. The interventions given in settings in LMICs only were included.

### 5.2 Search methods for identification of studies

Both published and unpublished studies were considered eligible for this review. There were no language restrictions. Relevant studies were sourced through literature searches of European and American bibliographic databases and general search engines. Grey literature was also sought. The databases systematically searched were Cochrane Central Registry of Controlled Trials, PubMed, EMBASE (HDAS), PsycINFO, ERIC (EBSCO), CINAHL, and Web of Science.

The search strategy first developed keywords, combined with Boolean operators, to assess potentially relevant articles on the PubMed database from 1970 until 2022 (see Supporting Information: Appendix 2). IPT was developed in the 1970s, and so the retrieval date started from there. Due to controlled vocabularies across each database, the search strategy terms were adjusted to compensate for different databases. The thesaurus of each database was checked to ensure no indexed terms were missed. Relevant thesaurus terms were added to the search strategy. The combination of keywords and thesaurus terms allowed the search strategy to be implemented.

The reference lists of relevant articles were hand‐searched and any potentially relevant studies that were identified were also retrieved and considered for inclusion. Furthermore, bibliographies and references of systematic reviews and meta‐analyses were examined to identify relevant studies in this field. Clinical trials were sought using the resource ClinicalTrials.gov. This website lists completed and ongoing clinical trials in the US and around the world.

Lastly, we conducted an advanced Google search where we used the same keywords used in the bibliographic database search. The first 20 pages of results were screened. In addition, we conducted the search again prior to submission to ensure we have collated the most recent data.

We anticipated that studies were likely to employ random assignment to treatment/comparison and pre‐test/post‐test design. In randomized controlled designs, the participants are randomly assigned to intervention or control groups, and in quasi‐experimental designs, where participants are allocated to groups using non‐random methods but steps are taken to control the confounders, for example, matching of groups, measures to ensure baseline equivalence for main outcomes, and so forth. It was expected that the treatment condition IPT would be compared to either usual/standard care or pharmacological therapy/other psychosocial therapies.

We planned to use the secondary outcomes separately in the summary of findings. It was also planned that if insufficient data would be reported in the primary studies, then, a narrative account of these outcomes would be provided. It was also anticipated that different studies may use different instruments for measuring the outcomes. Careful consideration was given to whether outcomes from different measures can be combined in a meta‐analysis.

#### 5.2.1 Electronic searches

A combination of keywords and thesaurus terms were used across the seven databases, with the exception of Web of Science, which does not contain thesaurus terms, and ERIC, which had no equivalent thesaurus entry for PPD. Databases were searched in 2020 and an updated search was employed in July 2022. All search terms, dates of searches, and results were recorded in Google Sheets. A total of 15,873 articles were found across all searched databases. Studies were uploaded into Covidence, where 6493 studies were removed as duplicates, leaving a total of 9380 eligible studies.

#### 5.2.2 Searching other resources

In addition to bibliographic database searches, we attempted to capture European grey literature through OpenGREY. The authors also contacted the experts in the field of IPT to identify any unpublished and ongoing studies. A list of the inclusion criteria for the review was sent to these experts along with the request for studies. We did not receive any studies conducted in LMICs from IPT experts.

Other resources were checked in April 2020, and again after the final result of the database search had been reached in November 2020 and again updated in July 2022. On all these occasions, no further eligible grey literature was found on OpenGREY. Furthermore, a keyword search on Google Scholar did not reveal any further studies that had not been found in the main literature search, and ClinicalTrials.gov did not yield any relevant ongoing trials.

### 5.3 Data collection and analysis

Data collection methods have been detailed below.

#### 5.3.1 Selection of studies

All the studies were uploaded into Covidence. In the first phase, five members of the review team independently reviewed the titles and abstracts to exclude irrelevant studies. Full texts of only those studies were retrieved, for which there was an agreement between the two reviewers. If there was a disagreement regarding the exclusion of a study, a third member of the review committee was called upon to resolve the disagreement.

The second phase involved a full‐text review of the chosen articles to determine eligibility based on inclusion criteria. Each paper was reviewed independently by two members of the review team. Here again, in case of any disagreement, a third member of the review team was consulted.

The rationale for the exclusion of the studies was documented for each full‐text article reviewed. Preferred Reporting Items for Systematic Reviews and Meta‐Analyses. PRISMA diagram was used to present the overall search and screening process (Moher, 2009).

#### 5.3.2 Data extraction and management

##### Details of study coding categories

The studies meeting the inclusion criteria were reviewed thoroughly and important information was extracted in tables. Two members of the review team extracted the relevant data from each included study into an evidence table. Data abstraction form was designed to gather pertinent information from the included studies such as study population characteristics, setting, intervention, comparators, study design, methods, results, and risk of bias. Two members of the team reviewed the data abstraction for completeness and accuracy. In case of any disagreement, the discussion was done to reach a consensus. Data were extracted based on the following coding categories: Publication Characteristics (Publication type, Author Details, Date, Settings of Study), participant information (age, country of residence, race/ethnicity, socioeconomic status), intervention (Type, mode, number of sessions and duration of follow up), comparison (type, mode, number of sessions and duration of follow up), Outcome measures (maternal outcome, couple outcomes, adverse effect outcomes), tools/instruments used for measurement for each outcome, results, risk of bias and data analysis.

#### 5.3.3 Assessment of risk of bias in included studies

For assessing the risk of bias in included studies of randomized trial designs, the original Cochrane Risk of Bias tool for the randomized designs (Higgins, 2011) was used. The risk of bias was assessed on seven domains; sequence generation, allocation concealment, blinding of participants and personnel, blinding of outcome assessment, incomplete outcome data, selective outcome reporting, and other issues. The risk of bias was judged as low, high, or unclear.

Two trained members of the team assessed the risk of bias in included studies independently. If studies did not report details to assess the validity of the design or study, it was reported that the risk of bias was unclear.

#### 5.3.4 Measures of treatment effect

The primary outcome of this review was a change in depression scores from pretest to posttest in intervention and control groups. The primary outcome effect size, that is, change in depression scores was reported as standard mean scores and standard deviation (SMD and SD) in the three studies included in the meta‐analysis. The treatment effect size was calculated as the difference between the standardized mean change in depression scores for the treatment and control groups as explained by Morris (2008).

#### 5.3.5 Unit of analysis issues

The unit of allocation in the included studies was individual participants. There were no cluster randomized trials included in this review. The time point for depression scores assessment post‐intervention in all the studies varied. Still, due to the small number of studies, the moderating effect of IPT at different follow‐up time points could not be evaluated. Furthermore, it was anticipated that comorbid conditions of postpartum women might have resulted in substantial heterogeneity in pooled effect, but, due to a very small number of studies, the effect due to co‐morbid conditions could not be analyzed.

#### 5.3.6 Dealing with missing data

Corresponding authors were contacted for the missing data in the studies. The data for one study could not be retrieved in spite of many requests to authors. This study was not included in the meta‐analysis.

#### 5.3.7 Assessment of heterogeneity

The heterogeneity was assessed by calculating *τ* ^2^, *χ* ^2^ and *I* ^2^ statistics. *τ* ^2^, which represents the between‐study variance, *χ* ^2^ assesses whether observed differences are due to chance alone and *I* ^2^ statistic, represents the percentage of total variation across studies that is due to heterogeneity rather than chance (Deeks, 2011). *I* ^2^ Statistics criteria of heterogeneity used were “0%–40%: might not be important, 30%–60%: may represent moderate heterogeneity, 50%–90%: may represent substantial heterogeneity and 75%–90%: may represent considerable heterogeneity” (Deeks, 2011).

#### 5.3.8 Assessment of reporting biases

In order to ascertain whether publication bias may be influencing the results, a number of grey literature sources were also searched for relevant material. Key authors were contacted for unpublished documents relevant to the research question. Trial registries were also searched (e.g., http://www.ctri.nic.in) from a range of countries.

#### 5.3.9 Data synthesis

Revman Web was used to conduct the meta‐analysis. Due to the limited number of studies available for inclusion, a fixed effects model was used to estimate the pooled effect sizes and corresponding 95% confidence intervals (CIs) for the primary outcome, that is, change in depression scores. Given the limited number of studies meeting the inclusion criteria and the inability to conduct subgroup or moderator analyses, a narrative synthesis was performed. The findings of each included study were summarized descriptively, highlighting the key results, methodological limitations, and potential implications for clinical practice.

#### 5.3.10 Subgroup analysis and investigation of heterogeneity

Subgroup analyses were planned based on potential sources of heterogeneity (e.g., study design, sample characteristics, and intervention variations). However, subgroup and moderator analyses could not be conducted due to the scarcity of eligible studies and inadequate data availability. So, the narrative description based on different variables has been presented in the results section.

#### 5.3.11 Sensitivity analysis

Sensitivity analysis was not conducted.

#### 5.3.12 Summary of findings and assessment of the certainty of the evidence

The summary of findings table has been presented along with the assessment of certainty of evidence. We used the GRADE (Grades of Recommendation, Assessment, Development, and Evaluation) approach for presenting the overall assessment of certainty of evidence (Atkins, 2004; Schünemann, 2008).

## 6 RESULTS

### 6.1 Description of studies

Description of the studies included and excluded is given below.

#### 6.1.1 Results of the search

**Figure 2:**
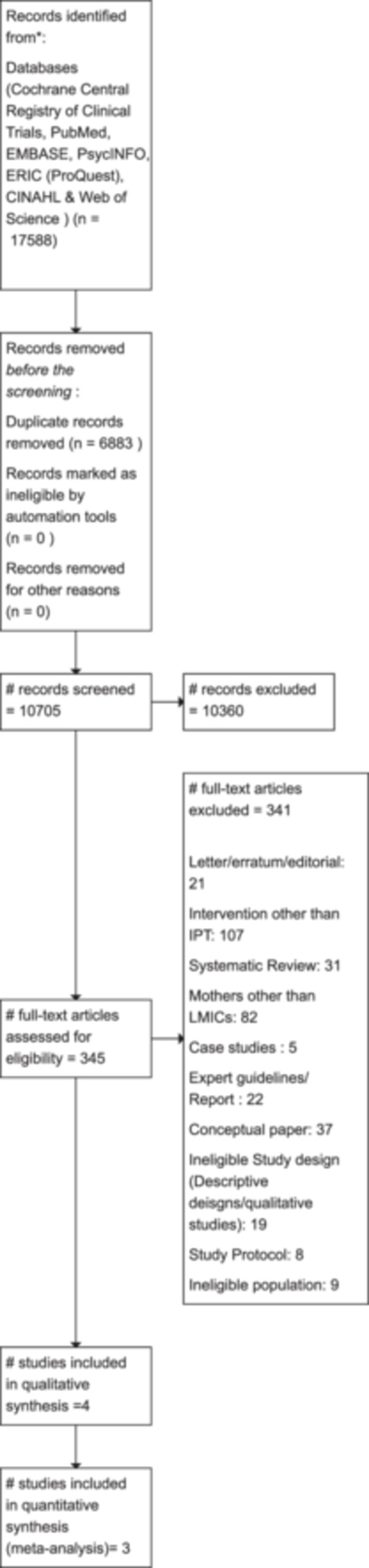
Prisma flow diagram.

Figure 2 summarizes the results of the search and screening for this review. Existing records in the databases; Cochrane Central Registry of Clinical Trials, PubMed, EMBASE, PsycINFO, ERIC ProQuest, CINAHL & Web of Sciences were identified through the search strategy and a total of 17,588 records were imported into Covidence. After the removal of duplicates, a total of 10,705 studies were screened based on title and abstract. Following this, 345 studies' full text were examined and 341 records were excluded [the reasons for exclusion were: Interventions other than IPT (107 records), mothers other than LMICs (82 records), ineligible study designs (19 records), systematic reviews (31 records), conceptual papers (37 records), patients, not PPD (9 records), commentary/letter/editorial/erratum (21 records), study protocols (8 records), case studies (5 records) and expert guidelines/reports: (22 records). Four (4) studies (Hajiheidari, 2013; Nusrat, 2016; Spelke, 2022; Yator, 2021) met the eligibility to be included in the review; however, one study Nusrat (2016) could not be included in the meta‐analysis due to the non‐availability of sufficient data.

#### 6.1.2 Included studies

Four studies were included in the final review based on the eligibility criteria with a total of 188 postpartum women. Details of included studies have been presented below:

##### Study designs

Three trials were feasibility trials; one study was an unmasked, un‐blinded randomized feasibility trial of IPT and antidepressant medication (ADM) on PPD and/or anxiety Spelke (2022). The second study was a pilot randomized trial to assess the acceptability, feasibility, and effectiveness of IPT on depression and antiretroviral therapy (Yator, 2021). The third study assessed the feasibility of IPT on maternal depression Nusrat (2016). And the fourth study was a RCT to assess the effectiveness of IPT on depression and marriage adaptation (Hajiheidari, 2013).

###### Participants

The four included trials had a total sample of 188 women suffering from PPD. One study included 34 women suffering from PPD, referred to health service and psychotherapy centers for counseling (Hajiheidari, 2013). The second study included 50 women aged above 18 years and with children below 3 years of age and experiencing mild to moderate depression (Nusrat, 2016). The study conducted by Yator included a total of 24 HIV+ women with PPD aged between 18 and 24 years and 6–12 weeks postpartum (Yator, 2021). Another study included 80 HIV+ women aged >18 years diagnosed with PPD 6–8 weeks postpartum from live birth (Spelke, 2022).

In all four studies, women were screened for depression using EPDS (Spelke, 2022; Nusrat, 2016; Yator, 2021; Hajiheidari, 2013). The study conducted by Hajiheidari (2013) also used the Beck II depression questionnaire for assessing the intensity of depression among women but did not report the scores of the Beck II depression questionnaire in the findings.

###### Setting

One study was conducted in the clinics (health services centers) and psychotherapy clinics in the Esfahan City of Iran (Hajiheidari, 2013). The second was conducted in a counseling center in Karachi, Pakistan (Nusrat, 2016). The study conducted by Yator (2021) provided the IPT in the two health centers (in one facility they used a spacious room of the health center's maternity building and in another facility, a social hall was used). The fourth study was conducted at two public postnatal clinics in Lusaka, Kenya (Spelke, 2022).

###### Intervention

The study conducted by Hajiheidari (2013), provided 10 weeks of marriage IPT to couples for PPD and marriage adaptation. Whereas, the other study used 10 sessions of IPT for PPD (Nusrat, 2016).

The study conducted by Yator (2021) employed Group Interpersonal therapy, that is, IPT‐G was provided by community health workers, who had undergone 2 days training on delivering the IPT‐G. The IPT‐G with treatment, as usual, was provided for 8 weeks for the intervention group.

The fourth study had two interventional arms: one group was provided with IPT and the second group was provided with ADM. All the participants were scheduled for 12 visits over 24 weeks (weekly visits for the first 4 weeks, biweekly visits for the next 8 weeks, and every 4 weeks thereafter). The IPT group received 11 sessions (in‐person or telephonic) from the trained study nurses. The intervention also included a structured program designed for the treatment of depression in patients with HIV (included sessions focussing on medication adherence, motherhood, poverty, HIV infection, social isolation, and stigma/discrimination) (Spelke, 2022).

###### Comparison

Three studies used the usual treatment as a comparison group (Hajiheidari, 2013; Nusrat, 2016; Yator, 2021) but one study conducted by Spelke (2022) compared IPT with ADM.

##### Outcome measures

###### Primary outcome

The primary outcome in all the included four studies was a change in depression scores (Hajiheidari, 2013; Nusrat, 2016; Spelke, 2022; Yator, 2021). All four studies used EPDS to assess depression among postpartum women.

###### Secondary outcome

The study conducted by Hajiheidari (2013) reported marriage adaptation and Nusrat (2016) reported Self‐esteem and quality of life as secondary outcomes. Whereas, Yator (2021) measured adherence to antiretroviral therapy and Spelke (2013) measured Global functioning and mental illness severity (Measured by Clinical Global Impression‐ Severity of Illness Score CGI‐S) and viral load as the secondary outcomes, as these two studies included HIV+ postpartum women as subjects. Only one study reported suicidal ideation in adverse outcomes and participant satisfaction regarding intervention (Spelke, 2022).

None of the studies reported adverse outcomes such as suicide attempts, suicides, number of hospitalization, duration of hospitalization, and lost workdays as outcomes. Only one study reported suicidal ideation and adverse drug reaction (Spelke, 2022). Furthermore, we could not find any other maternal outcomes reported in the included studies such as maternal‐infant bonding/attachment, social support, postpartum adjustment, social adjustment, and transition back to work. As for couple outcomes; marriage adaptation was reported by one study (Hajiheidari, 2013), and sexual interest was also not reported by any of the included trials.

#### 6.1.3 Excluded studies

A total of 341 studies were excluded. The reasons for the exclusion of studies were mothers from other than LMICs, interventions other than IPT (psycho‐education, psychoanalysis, CBT, etc.), systematic reviews, conceptual papers, case studies, and commentaries/letters/editorials.

### 6.2 Risk of bias in included studies

**Figure 3:**
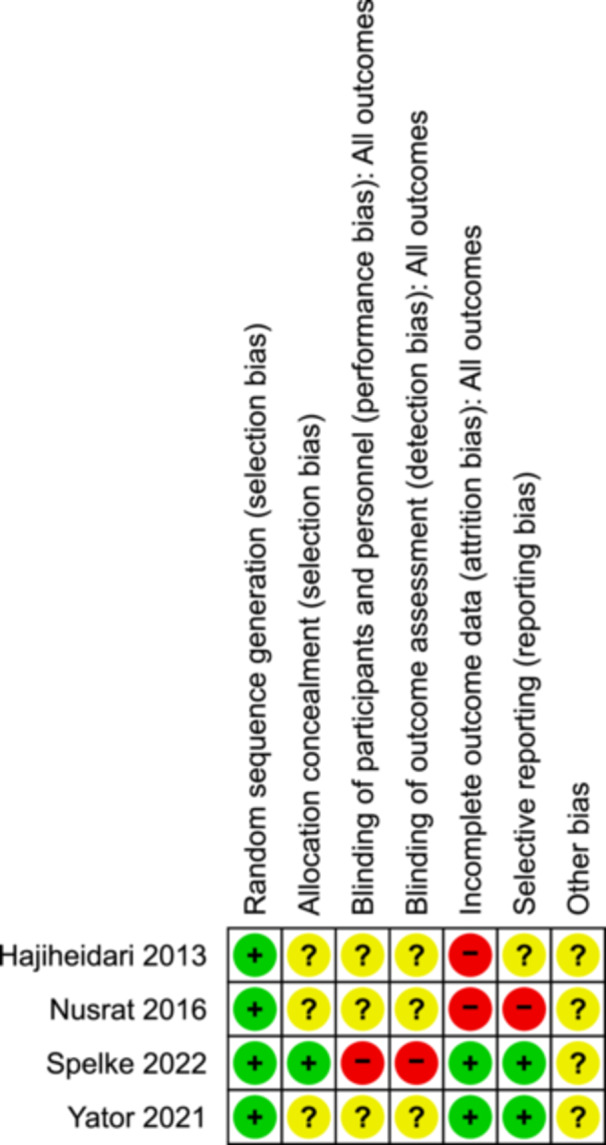
Risk of bias summary.

The risk of bias in included studies was assessed using the revised Cochrane Risk of Bias tool for the randomized tool (RoB 2.0) (Higgins, 2011). The risk of bias was assessed on seven domains; sequence generation, allocation concealment, blinding of participants and personnel, blinding of outcome assessment, incomplete outcome data, selective outcome reporting, and other issues. The risk of bias was judged as low risk, high risk, or unclear risk (Figure 3).

#### 6.2.1 Allocation

All four studies reported random allocation of subjects to experimental and control groups so a low risk of bias was judged for these studies in relation to sequence generation and allocation (selection bias) (Hajiheidari, 2013; Nusrat, 2016; Spelke, 2022; Yator, 2021).

#### 6.2.2 Blinding

The blinding of participants and researchers was not reported in the three studies. Therefore, the performance, as well as detection bias, was judged as unclear in the studies conducted by Hajiheidari (2013), Nusrat (2016), and Yator (2021). The fourth study was an unmasked and unblinded feasibility study, so the risk of bias was judged as high (Spelke, 2022).

#### 6.2.3 Incomplete outcome data

The attrition bias was judged as unclear as both studies did not report any information on attrition in the post‐test and follow‐up. One study did not report the timings of the post‐test after the intervention (Hajiheidari, 2013), whereas the second study assessed the outcome measures at 3 months and 6 months (Nusrat, 2016). The studies conducted by Spelke (2022) and Yator (2021) reported complete information on recruitment and attrition so the risk of bias was judged as low.

#### 6.2.4 Selective reporting

The study conducted by Hajiheidari (2013) reported all the outcome measures, that is, depression and marriage adaptation, so reporting bias for this study was judged as low. The second study did not report all the measures except depression at a follow‐up duration of 3 months and 6 months, so the reporting bias was judged as high (Nusrat, 2016). The other two studies reported all the outcome measures so the risk of bias was judged as low (Spelke, 2022; Yator, 2021).

#### 6.2.5 Other potential sources of bias

No other major source of risk of bias was noted in all four studies.

### 6.3 Effects of interventions

#### 6.3.1 Primary outcome

The effect size of studies was the mean difference in depression scores from the pre‐test to post‐test in the treatment and control groups. Included four studies assessed the final depression scores at different time points. One study assessed the final depression scores at 10 weeks and found a significant reduction in scores in the IPT group at 10 weeks in comparison to the control group which received the usual treatment (SMD: 8.80, 95% CI (11.13, 6.48)) (Hajiheidari, 2013). The second study conducted by Nusrat (2016) assessed the depression scores at 3 months and 6 months and reported a significant reduction in depression scores at 3 months (*p* < 0.000) and 6 months (*p* < 0.000) in the IPT group when compared to the usual treatment group. The study conducted by Yator (2021) assessed the depression scores at baseline, 8 weeks, 16 weeks, and 24 weeks. They reported significantly reduced depression scores in the IPT group at 8 weeks (SMD: 0.94, *t*: −2.29, df: 22) and at 16 weeks (SMD: 0.42, *t*: −1.01, df: 22) and at 24 weeks (SMD: 0.24, *t*: −0.55, df: 20) as compared to waitlist usual treatment group. Another study assessed the depression scores at 11 weeks and reported significantly reduced depression scores in the IPT group as compared to the ADM group (SDM: 2.44 (0.13, 4.74)) (Spelke, 2022).

##### Meta‐analytic estimates

###### Depression scores: IPT versus other interventions (Figure 4, Analysis 1.1)

**Figure 4:**
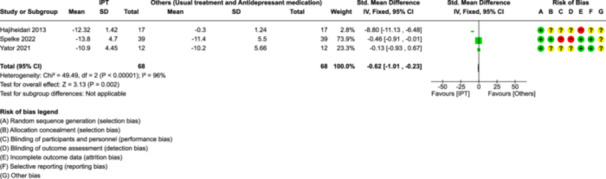
Meta‐analyses: Depression Scores: IPT versus Other Interventions.

Data from three studies were used to calculate the pooled effect of IPT on depression scores (Hajiheidari, 2013; Spelke, 2022; Yator, 2021). One study conducted by Nusrat (2016) could not be included in the meta‐analysis due to insufficient data. Depression scores reduced significantly in the IPT group as compared to other interventions (Usual treatment or ADM) (SMD: −0.62, 95% CI (−1.01, −0.23), 3 studies, *n* = 136). Substantial heterogeneity was found (*χ* ^2^: 49.49; df = 2 [*p* < 0.00001]; *I* ^2^ = 96%). The heterogeneity can be attributed to the small sample size of individual studies, population differences (Postpartum women with and without HIV), the difference in comparison intervention, and so forth. Although a large effect size was found in three studies (Hajiheidari, 2013; Spelke, 2022; Yator, 2021), due to substantial heterogeneity, concern remains about whether IPT is effective in reducing depression scores compared to other treatment options.

#### 6.3.2 Secondary outcomes

##### Couple outcomes

###### Marriage adaptation

One study assessed the marriage adaptation by using the revised double adaptive scale and reported significant improvement in the marriage adaptation in the couple/paired IPT group as compared to the usual treatment group (*F*: 485, *p* < 0.001, *n* = 34) (Hajiheidari, 2013).

##### Adverse events

###### Adverse drug reaction

The adverse drug reaction outcome variable was reported in only one study. The study conducted by Spelke (2022) included HIV+ postpartum mothers and compared the adverse drug reactions in the IPT group and the ADM group. Seven patients reported adverse drug reactions in the ADM group with no participant reporting adverse drug reactions in the IPT group (RR 15.0, 95% CI (0.89, 254), *p* = 0.06, *n* = 78).

###### Suicidal ideation

Only one study reported suicidal ideation (Spelke, 2022) and reported two participants having suicidal ideation in the IPT group and one in the ADM group (RR 0.50, 95% CI (0.05, 5.30), *p* = 0.56, *n* = 78).

###### Severe depression

The study conducted by Spelke (2022) reported that none of the participants in the IPT group had severe depression whereas one participant in the ADM group had severe depression (RR 3.00, 95% CI (0.13, 71.5), *p* = 0.50, *n* = 78).

##### Intervention satisfaction

Only one study surveyed the participants for satisfaction in relation to the intervention. The interventions employed in the study were IPT and ADM. All the participants (100%, *n* = 78) reported that they were happy to be in the study and also reported that they felt better after the intervention in both the groups; IPT and ADM (Spelke, 2022).

#### 6.3.3 Subgroup analysis

Subgroup analysis was explored, but due to the very small number of studies, subgroup analysis was not possible so a narrative description has been given below:

IPT was reported to be effective in reducing postnatal depression scores significantly as compared to treatment as usual (TAU) as reported by Hajiheidari (2013); Nusrat (2016); Yator (2021) and ADM reported by Spelke (2022). Furthermore, IPT given for varied durations ranging from 8 to 12 sessions was reported to be effective in reducing postnatal depression scores among women.

IPT was delivered by psychologists in one study (Hajiheidari, 2013), nurses in another study (Spelke, 2022), and community health workers in the third study (Yator, 2021). Irrespective of different providers, the IPT was found to be effective in reducing depression scores. One study did not mention who were the IPT providers (Nusrat, 2016).

Out of four studies, two included HIV+ postpartum women. IPT was also found to be effective in reducing depression among HIV+ Postnatal women (Spelke, 2022; Yator, 2021).

#### 6.3.4 Narrative description of studies

The narrative description of the results of all four studies has been presented below.

The study conducted by Hajiheidari (2013) recruited 34 PPD women and allocated them randomly to either 10 weeks paired IPT or usual treatment. Assessments were conducted using EPDS to assess depression and RDAS for marriage adaptation. The study findings reported that 10 weeks of pair‐IPT sessions were effective in reducing depression and improving marriage adaptation significantly in comparison to the control group (who received the usual treatment). Furthermore, *F*‐coefficients were computed after omitting the effect of the pre‐test and reported a significant difference between the experimental and control groups in terms of depression and marriage adaptation post‐test scores. The methodology of the study is quite weak as the authors did not explain the data collection methods and interventions clearly. The sample size of this study was small.

Another study conducted by Nusrat (2016), reported using 10 sessions of group IPT for the experimental group and TAU for the control group. Assessments were conducted using the EPDS, Rosenberg's Self‐esteem Scale, and EuroQol −5D to measure depression, self‐esteem, and health‐related quality of life respectively. The assessments were done at baseline, 3 months, and 6 months. The findings were reported only for depression and it was reported that mean scores of depression reduced significantly at 3 months and 6 months in the IPT group in comparison to the TAU group. However, this study's full text could not be retrieved so this information is based on the findings reported in the conference abstract only. The abstract did not report the outcome measures of self‐esteem and quality of life of participants.

The third study conducted by Yator (2021) recruited 24 HIV+ mothers and allocated them to either an intervention (IPT) group or a waitlist group. The intervention group received IPT sessions for 8 weeks administered by community health workers and the waitlist group received routine services at PMCTC (“Preventing Mother to Child Transmission” Center) including educational sessions on sexual and reproductive health, male partner involvement, child and maternal nutrition, and child immunization. The waitlist group was offered the intervention after the first 8 weeks and was followed for another 8 weeks. and the intervention group was also followed for a cumulative 16 weeks. For any marital conflict, the male partners were also called for the couple's session taken by community health workers. At 8 weeks follow‐up, the depression scores (EPDS scores) reduced significantly greater in the IPT group than in the waitlist group. Furthermore, the depression scores reduced gradually from week 1 to week 16 and reduced significantly more in the IPT group than in the waitlist group. There was no significant difference reported in treatment adherence. This study was a pilot randomized trial so the sample size of the study was very small and accordingly the generalizability of the findings is a concern.

The fourth study conducted by Spelke (2022), reported using two arms interventions: one group was given IPT and another was given ADM. The sample consisted of 78 HIV+ women receiving antiretroviral therapy (ART). The participants were randomly allocated to either the IPT group or the ADM group. All participants were scheduled for 12 visits over 24 weeks, with weekly visits for the first 4 weeks, biweekly visits for the next 8, and every 4 weeks thereafter. Study visits were performed by trained research staff in a dedicated study space. Participants in the IPT group received 11 sessions from trained staff nurses, followed by a structured program adapted from the evidence‐based Mental Health Integration Program, designed for the treatment of depression in patients with HIV in South Africa with sessions on medication adherence, motherhood, poverty, HIV infection, social isolation, and stigma/discrimination as they relate to depression. The staff nurses who provided the IPT were trained in IPT by undergoing a 1‐week in‐person workshop and virtually they were supervised by a licensed psychologist during the whole period of study. The participants in the ADM group were instructed to self‐administer SSRI, that is, 25 mg Sertraline self‐administer orally daily from the day of recruitment. An evidence‐based treatment algorithm was followed to titrate the dosage weekly with 25 mg increments until the desired effect was achieved which was assessed by EPDS and CGI‐S scores. The side effects of the medication were assessed during each visit. The findings reported a significantly greater reduction in depression scores (EPDS scores) in the IPT group as compared to the ADM group, whereas (clinical global impression‐severity scores) CGI‐S scores did not differ significantly across both groups. two participants in the IPT group were reported to have suicidal ideation. Patient satisfaction was significantly higher in the IPT group as compared to the ADM group. The strengths of this study are the random allocation of subjects in groups but the authors did not report the blinding of participants and assessors.

##### Sensitivity analysis

Due to a very small number of studies, sensitivity analysis was not possible to conduct.

#### 6.3.5 Assessment of reporting biases

**Figure 5:**
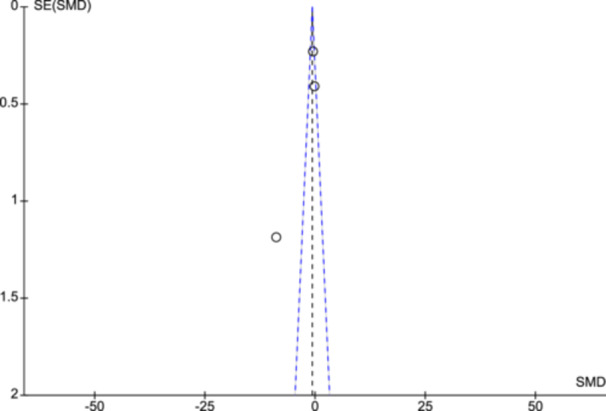
Funnel plot.

Figure 5 displays funnel plots on depression score changes (IPT vs. Usual treatment). Due to only three studies, the interpretation of publication bias cannot be made as asymmetry cannot be evident and may simply be due to chance.

## 7 DISCUSSION

### 7.1 Summary of main results

The primary objective of this review was to evaluate the efficacy of IPT in addressing depression among postpartum women residing in LMICs. The review encompassed four RCTs. In total, 188 postpartum mothers participated in these studies.

This review specifically focused on studies conducted exclusively on postpartum mothers residing in LMICs. The included studies encompassed two conducted in African countries, one in Pakistan, and one in Iran. In terms of the interventions utilized, two studies employed group IPT, while one study utilized couple IPT. As for the comparison interventions, three studies included TAU, while one study compared IPT with ADMs.

A meta‐analysis was conducted by pooling the findings of three studies to evaluate the effectiveness of IPT in comparison to other therapy options, such as usual treatment or ADM, for PPD. The results indicated a significant reduction in depression scores among the IPT group when compared to the other treatment options.

Additional findings reported in the studies included improvements in marriage adaptation, as observed in one out of the four studies. Another study reported enhancements in global functioning and reduced the severity of mental illness. Moreover, one study reported higher satisfaction levels with the IPT intervention.

Two studies specifically included postpartum mothers with HIV and investigated the impact of IPT on antiretroviral adherence compared to the control group. The findings indicated no significant difference between the IPT group and the control group in terms of antiretroviral adherence.

Due to the limited number of studies available, subgroup analysis for various covariates, such as HIV status, mode of IPT, provider of IPT, and duration of IPT, could not be conducted. The meta‐analysis revealed high heterogeneity among the studies, which can be attributed to variations in participant characteristics, including HIV status, different modes of IPT, small sample sizes of the studies, and different providers of IPT intervention. In future reviews focusing on LMICs, it is recommended to consider the potential impact of these moderators on the outcomes in the meta‐analysis.

### 7.2 Overall completeness and applicability of evidence

The present review adhered to the systematic review process and employed rigorous methods. Following a comprehensive systematic search strategy, including title screening and full‐text screening, only four studies were deemed eligible for inclusion. While the pooled effectiveness results of the included studies indicated a reduction in PPD following treatment with IPT, it is important to note that the evidence cannot be confidently applied to LMICs due to the low certainty of the evidence of the included studies. Moreover, due to incomplete information in one study, only three studies could be combined for meta‐analysis. The studies did not provide details about the analysis plan or protocol, thus making it difficult to ascertain reporting bias. Additionally, although all the studies reported random allocation to treatment and control groups, blinding was not implemented, leading to a high risk of bias in the studies. Furthermore, the lack of subgroup analysis prevented an examination of the impact of confounding variables, which could significantly influence the effectiveness of the intervention.

### 7.3 Quality of the evidence

The overall quality of evidence generated by this review is considered weak primarily due to the limited number of included studies, with only four studies meeting the eligibility criteria. Furthermore, the meta‐analysis could only be conducted on three of these studies, further reducing the available evidence. Within this limited pool of studies, two were identified as low‐quality studies with significant limitations.

One of the studies, conducted by Hajiheidari (2013), lacked crucial information regarding allocation concealment, blinding of participants and researchers, and attrition rate. These omissions introduce potential biases and undermine the reliability and validity of the study's findings. The second study, conducted by Nusrat (2016), was a short communication piece, providing limited details that prevented firm conclusions from being drawn. Despite attempts to obtain complete information from the researchers, the missing data rendered the study of low quality. Among the remaining two studies (Spelke, 2022; Yator, 2021), which reported comprehensive methodologies and findings, it is important to note that they specifically focused on postpartum women with HIV+. This specific subgroup inclusion contributes to the observed heterogeneity among the studies, which may impact the generalizability of the results.

Additionally, the risk of bias in the included studies was deemed high due to several factors. The absence of blinding and allocation concealment in the treatment and control groups introduced performance bias and potentially influences the reported outcomes. Furthermore, the lack of information on the protocol or plan of analysis in the included studies hindered the assessment of reporting bias, thereby limiting the reliability of the findings.

Due to the limited number of studies available for inclusion, conducting subgroup analyses to examine the effects of confounding variables such as HIV status, type of IPT, duration of IPT, IPT providers, and so forth, was not feasible. This limitation prevents a comprehensive understanding of the potential impact of these variables on the effectiveness of IPT in reducing depression scores among postpartum women in LMICs. Moreover, notable heterogeneity was observed in the pooled effect estimates of IPT on depression scores when compared to usual treatment or ADM.

Given these limitations, it is essential to exercise caution when interpreting the findings of this systematic review. The high risk of bias, lack of blinding, absence of a protocol or plan of analysis, unavailability of subgroup analyses, and high heterogeneity among studies collectively contribute to the overall uncertainty in the quality of evidence.

To improve the robustness of future research in this area, it is recommended that studies employ rigorous methodologies, including blinding, detailed protocols, and appropriate analyses, to minimize bias and enhance the overall quality of evidence on the effectiveness of IPT on reduction of depression scores in postpartum women in LMICs.

### 7.4 Potential biases in the review process

The authors of this review took necessary measures to minimize bias throughout the study selection and analysis process. Two independent reviewers conducted the eligibility assessment, data extraction, and quality assessment of the included studies. Any discrepancies in study eligibility were resolved through thorough discussion among the reviewers. However, it is important to acknowledge that subjectivity may exist during the screening process, introducing the possibility of unintentional biases.

Moreover, the four included studies exhibited variations in participant characteristics, with two studies specifically focusing on postpartum mothers with HIV. However, the pooled effectiveness analysis included three studies that encompassed postpartum mothers both with and without HIV. The inclusion of HIV status as a potential confounding variable that could affect depression scores introduces limitations to the pooled effectiveness analysis. Additionally, there is a possibility of high heterogeneity among the studies regarding the pooled effect of IPT on depression when compared to other treatment options. These factors pose potential sources of bias.

Despite the aforementioned potential biases, it is noteworthy that all the studies consistently indicated an improvement in depression scores within the IPT group as compared to other treatment options in LMICs.

### 7.5 Agreements and disagreements with other studies or reviews

Since this was the first review on the topic in LMICs, we could not conduct any comparative assessment with other reviews conducted on women with PPD in LMICs. But there are systematic reviews undertaken in the developed world and evidence from our review is consistent with these systematic reviews. All the reviews consistently reported IPT as effective in reducing depression scores (Dennis, 2007; Dennis, 2013; Miniati, 2014; Sockol, 2018). Dennis and Hodnett conducted a review of psychosocial and psychological interventions for PPD, they included only one trial which assessed the efficacy of IPT and reported that depressive symptomatology decreased at the post‐treatment assessment (Dennis, 2007). Miniati et al. (2014) reported the evidence from 11 trials and found that IPT resulted in clinical improvement and complete recovery in some cases. Furthermore, they also reported that IPT alone or in combination with antidepressant therapy was effective in shortening the recovery time and prolonging the remission period. But they presented the narrative analysis only and did not report any pooled effects of these findings (Miniati, 2014). The evidence from the present review also suggests that IPT may be effective in reducing depression in postpartum women but due to high heterogeneity among the three studies included in the meta‐analysis, definitive conclusions about the effectiveness of IPT for PPD in LMICs cannot be made.

## 8 AUTHORS' CONCLUSIONS

### 8.1 Implications for practice

This review's findings suggest that IPT may be effective in reducing depression in postpartum mothers residing in LMICs. It is reported by previous research studies that PPD is more prevalent in LMICs as compared to upper‐income countries (WHO, 2016b; Fisher, 2012; Roddy, 2023). Furthermore, a number of complications are associated with PPD such as high mortality due to suicides among postpartum women, and malnutrition, stunting of growth, higher hospitalizations, and higher incidences of infections among infants (Fisher, 2012; Stewart, 2008). IPT is recommended as a first‐line treatment option for PPD but it is very important to have strong evidence on the efficacy of IPT on PPD. Moreover, various factors such as local contexts and cultural appropriateness are needed to be explored before the implementation of this therapy in actual settings. IPT has been reported to be effective in reducing depression in comparison to other psychosocial and pharmacological therapies in previous reviews and meta‐analyses conducted in developed nations (Miniati, 2014; Sockol, 2018) but this review conducted to find the efficacy of IPT for PPD in LMICs could not reach the definite conclusion due to the lack of well‐designed investigations, high heterogeneity, small sample size, varying settings, variable population, and so forth. This points towards the need for more rigorous and continuous research and implementation efforts and integration of mental health services into maternal healthcare services in the context of low‐resource settings.

### 8.2 Implications for research

Given the very small number of studies found eligible in the review, the authors recommend conducting more primary studies in this area in LMICs. Furthermore, evidence could not be found concerning the effectiveness of IPT on maternal outcomes such as mother‐infant bonding/attachment, anxiety, social support, postpartum adjustment, social adjustment, transition back to work, and adverse outcome measures such as suicide attempts, suicides, number of hospitalizations, duration of hospitalization and lost workdays. Therefore there is a need for further research on the effectiveness of IPT on these secondary outcomes. Only one study could be found that reported improvement in marriage adaptation in terms of couple outcome but the evidence cannot be conclusive given the low quality of the study. More randomized trials with bigger sample sizes, rigorous methodology, and reduced risk of bias need to be conducted to strengthen the evidence.

## DATA AND ANALYSES

Comparison 1

IPT versus Others (Usual treatment or ADM)

**Table:** 

Outcome or subgroup title	No. of studies	No. of participants	Statistical method	Effect size
1.1 Depression Scores (IPT vs. Others)	3	136	Std. Mean Difference (IV, Fixed, 95% CI)	−0.62 [−1.01, −0.23]

## WHAT'S NEW

**Table:** 

Date	Event	Description
30 December 2019	Amended	Protocol Resubmission
14 June 2019	Amended	Protocol
14 June 2019	Feedback has been incorporated	Protocol

## CONTRIBUTIONS OF AUTHORS

**Roles and responsibilities**

Content: Bandana Bisht, Harmeet Kaur

Systematic review methods: Harmeet Kaur, Bandana Bisht, Manmeet Kaur, Aaron Worsley, Obrey Alexis, Denny John

Meta‐analysis: Harmeet Kaur

Information retrieval: Aaron Worsley

## DECLARATIONS OF INTEREST

No potential conflicts are involved for any of the authors of this review.

## SOURCES OF SUPPORT

### Internal sources

- Campbell Collaboration Social Support Group, UK (Support provided for Covidence software)

### External sources

- None, Other

No any external sources of support.

## DIFFERENCES BETWEEN PROTOCOL AND REVIEW

Our review is based on our published protocol (Kang, 2020). We used the fixed effects model to assess the pooled effect rather than the random effects model as planned in the protocol due to the very small number of eligible studies. We planned to assess the pooled effect of IPT for secondary outcomes; maternal outcomes such as maternal‐infant bonding, anxiety, social support, postpartum adjustment, social adjustment and transition back to work and adverse outcomes such as suicides, suicidal attempts, number of hospitalizations in couple outcomes: sexual interest. But abovementioned outcomes were not found to be reported in the included studies. Furthermore, due to the small number of studies included in this review, sub‐group analysis in terms of mode of delivery of IPT, number of sessions, and providers of IPT could not be conducted as planned in the protocol. Sensitivity analysis also could not be conducted due to very few studies included in the meta‐analysis.

## NOTES

Kang, HK, John, D, Bisht, B, Kaur, M, Alexis, O, Worsley, A. PROTOCOL: Effectiveness of interpersonal psychotherapy in comparison to other psychological and pharmacological interventions for reducing depressive symptoms in women diagnosed with postpartum depression in low and middle‐income countries: A systematic review. *Campbell Systematic Reviews*. 2020; 16:e1074. https://doi.org/10.1002/cl2.1074

## Supporting information

Supporting information.

Analysis 1.1 Depression Scores: IPT vs. Other Interventions.
