# Supplementary material for: Effectiveness of interpersonal psychotherapy in comparison to other psychological and pharmacological interventions for reducing depressive symptoms in women diagnosed with postpartum depression in low‐ and middle‐income countries: A systematic review
Source: Campbell Syst Rev. 2024 Apr 21;20(2):e1399. doi: 10.1002/cl2.1399 (PMC11032640; doi:10.1002/cl2.1399)
Supplement: Supplementary file 1 — Supporting information. [file CL2-20-e1399-s001.docx]

Characteristics of studies

Characteristics of included studies [ordered by study ID]

Hajiheidari 2013

| ***Study characteristics*** | | |
| --- | --- | --- |
| Methods | Pretest-posttest Randomized controlled study design | |
| Participants | Women diagnosed with PPD (IPT: n=17, Control: n=17)  Age in years: (IPT group) Mean (SD): 27.2 (2.3)  Age in years: (Usual treatment group) Mean (SD): 28.51 (12.3) years.  Inclusion Criteria: lack of personality and psychological disorders, no background for self-killing actions or thoughts, no abuse of alcohol or drugs, disuse of anti-depressive drugs, lack of reception of any other psychological treatment for present depression, no background of basic or dipolar depression, and husbands’ participation in therapy sessions.  Exclusion Criteria: Not mentioned | |
| Interventions | Experimental group: 10-week couple/pair interpersonal psychotherapy sessions delivered by psychologists  Control Group: Usual treatment. There was no further information given for usual treatment. | |
| Outcomes | Depression assessed by EPDS  Marriage Adaptation assessed by Revised double adaptive score (RDAS) | |
| Notes | Setting: Counseling Centers and clinics in Esfahan City, Iran  Ethical Approval: No information.  Study Period: April -June 2012  Declaration of Interest: None  Funding: None | |
| ***Risk of bias*** | | |
| **Bias** | **Authors’ judgement** | **Support for judgement** |
| Random sequence generation (selection bias) | Low risk | The study reported random allocation of subjects in the intervention and control group |
| Allocation concealment (selection bias) | Unclear risk | The study did not report the allocation concealment. |
| Blinding of participants and personnel (performance bias)  All outcomes | Unclear risk | No information about blinding of participants was reported. |
| Blinding of outcome assessment (detection bias)  All outcomes | Unclear risk | The study did not report anything about the process of blinding of outcome assessment. |
| Incomplete outcome data (attrition bias)  All outcomes | High risk | The discrepancy in the number of participants mentioned in the methodology and results section |
| Selective reporting (reporting bias) | Unclear risk | . |
| Other bias | Unclear risk | . |

Nusrat 2016

| ***Study characteristics*** | | |
| --- | --- | --- |
| Methods | Randomized Controlled Trial | |
| Participants | A total of 50 mothers aged 18 years and above with children below 3 years of age experiencing mild to moderate depression | |
| Interventions | Experimental Group: 10 sessions of group Interpersonal Psychotherapy  Comparison Group: Treatment as Usual (No further information provided on treatment to the control group) | |
| Outcomes | Depression assesed by EPDS  Self esteem assesed by Rosenberg Self Esteem Scale  Health related quality of life assessed by Euro-Qol-5D | |
| Notes | Setting: Not mentioned. The study was conducted in Karachi, Pakistan  Ethical Approval: No information  Funding: No infomation  Study Period: No information  Declaration of Interest: No Information | |
| ***Risk of bias*** | | |
| **Bias** | **Authors’ judgement** | **Support for judgement** |
| Random sequence generation (selection bias) | Low risk | The study reports the random selection of subjects in the intervention and control group, so the selection bias was reported as low. |
| Allocation concealment (selection bias) | Unclear risk | The study did not report how the subjects were assigned to intervention and control group. |
| Blinding of participants and personnel (performance bias)  All outcomes | Unclear risk | The information on blinding of particiants and personnel is missing in the study. |
| Blinding of outcome assessment (detection bias)  All outcomes | Unclear risk | The study did not report blinding of outcome assessment. |
| Incomplete outcome data (attrition bias)  All outcomes | High risk | The data on outcome variables is incomplete. The findings related to outcome variables; quality of ife and self esteem have not been reported. |
| Selective reporting (reporting bias) | High risk | The authors reported the results of EPDS scores only, the information on self esteem and quality of life are missing |
| Other bias | Unclear risk | . |

Spelke 2022

| ***Study characteristics*** | | |
| --- | --- | --- |
| Methods | Un-masked, Un-blinded, randomized feasibility trial of interpersonal psychotherapy (IPT) versus antidepressant medication (ADM) | |
| Participants | Postpartum HIV-positive women with depression and/or anxiety who were receiving ART in Lusaka.  Sample Size: IPT (n = 40) and ADM (n = 40)  Age in Years Mean (SD): 29.7 (5.4) (IPT: 30.0 (5.3); ADM: 29.5 (5.5)  Inclusion criteria: (1) ≥18 years of age; (2) documented HIV-1 infection; (3) current ART treatment; (4) 6–8 weeks postpartum from live birth;(5) diagnosis of major depression and/or generalized anxiety on MINI; (6) willingness to provide informed consent; and  (7) willingness to adhere to study procedures.  Exclusion Criteria: (1) active suicidal thoughts during pre-screening (defined as thoughts of suicide with a specific plan); (2) known or suspected allergy or contraindication to selective serotonin reuptake inhibitors (SSRIs); (3) history of ADM use within 12 months; or (4) any other condition (social or medical) which, in the study staff’s opinion, would make trial participation unsafe or complicate data interpretation. | |
| Interventions | All participants were scheduled for 12 visits over 24 weeks, with weekly visits for the first 4 weeks, biweekly visits for the next 8, and every 4 weeks thereafter.  Experimental Group: Interpersonal psychotherapy (IPT). Participants randomized to IPT received up to 11 therapy sessions from trained study nurses. Sessions followed a structured program adapted from the evidence-based Mental Health Integration Programme, designed for the treatment of depression in patients with HIV in South Africa, and included sessions explicitly focused on medication adherence, motherhood, poverty, HIV infection, social isolation and stigma/discrimination as they relate to depression. Research nurses completed an intensive 1-week, in-person workshop on the treatment program and received 1 hour of virtual super- vision from a licensed psychologist weekly throughout the  study.  Control Group: Antidepressant medication (ADM). Participants randomized to ADM were instructed to self-administer a daily oral SSRI (25 mg sertraline) from the day of randomization. Following an evidence-based treatment algorithm. Dosage was titrated weekly, in 25 mg increments, until treatment response was achieved based on EPDS and CGI-S scores. | |
| Outcomes | Depression scores assessed by EPDS  Viral Load  Global functioning and mental illness severity assessed by CGI-S (Clinical Global Impression Severity) | |
| Notes | Setting: Two public postnatal clinics in Lusaka, Zambia: Chawama First-level Hospital and Kamwala District Health Clinic.  Ethical Approval: The trial was registered with clinicaltrials.gov (NCT04094870) and approved by the University of North Carolina Institutional Review Board (17-3411) and the University of Zambia Research Ethics Committee (011-11-18).  Funding: This study was supported by a grant from the National Institute of Mental Health (PI: Stringer, E.; NIMH 1R21MH115806).  Study Period: Between 29 October 2019 and 8 September 2020  Declaration of interest: None | |
| ***Risk of bias*** | | |
| **Bias** | **Authors’ judgement** | **Support for judgement** |
| Random sequence generation (selection bias) | Low risk | Participants were randomized with equal probability to either ADM or IPT using an electronic system (REDCap) with sealed paper envelopes as a backup. |
| Allocation concealment (selection bias) | Low risk | A statistician from the UNC Center for AIDS Research Biostatistics Core not associated with the study designed the randomization scheme using random permuted blocks of varying sizes. |
| Blinding of participants and personnel (performance bias)  All outcomes | High risk | The authors conducted an unmasked and unblinded feasibility study. |
| Blinding of outcome assessment (detection bias)  All outcomes | High risk | The authors conducted an unmasked and unblinded feasibility study. |
| Incomplete outcome data (attrition bias)  All outcomes | Low risk | The outcome data is complete and informtaio on attrition has been provided in the study. |
| Selective reporting (reporting bias) | Low risk | All the outcome measures are reported completely. |
| Other bias | Unclear risk | No other bias |

Yator 2021

| ***Study characteristics*** | | |
| --- | --- | --- |
| Methods | The participants were tandomly assigned to IPT-G and waitlist group. The IPT-G was administred for 8 weeks | |
| Participants | Twenty-four HIV+ mothers aged 18-24 years and 6-12 weeks postpartum were included in the study.  Intervention group (N=12) and waitlist group (N=12).  Mean postdelivery duration: 8.08 weeks  Median age of participants: 23.0 years (interquartile range=3). | |
| Interventions | Experimental group: IPT-G (Group Interpersonal Therapy) was administered for 8 weeks to the intervention group. IPT was delivered by community health workers.  The waitlist group entailed routine services offered at PMTCT (“Preventing mother-to-child transmission) clinics at the selected health centers.  After the first 8 weeks, the intervention was offered to the waitlist control group for 8 weeks and the waitlist group followed up for another 8 weeks. Thus, the intervention group was monitored cumulatively for 16 weeks from the time IPT-G was completed. | |
| Outcomes | Change in Depression score assessed by EPDS (Edinburgh Postnatal Depression Score)  Compliance with antiretroviral therapy assessed by The Center for Adherance Support Evaluation Adherance Index. | |
| Notes | Setting: Kangemi and Kariobangi health centers in Nairobi, Kenya.  Funding: One of the authors received grant funding from the Thrasher Foundation and funding from the University of Washington, International Maternal Pediatric Adolescent AIDS Clinical Trials (IMPAACT), and UpToDate.  Ethical Approval: This pilot trial was reviewed and approved by the Kenyatta National Hospital–University of Nairobi Ethics and Research Committee (approval no. P97/02/2018).  Study Duration: Between August 2018 and July 2019  Declaration of Interest: None | |
| ***Risk of bias*** | | |
| **Bias** | **Authors’ judgement** | **Support for judgement** |
| Random sequence generation (selection bias) | Low risk | Quote: “Twenty-four pieces of paper were allocated binary numbers, either 1 (for the intervention group) or 2 (for the waitlist control group). The participants were then asked to choose a folded piece of paper; those with the number 1 formed the intervention group (N=12), and the remainder formed the waitlist control group (N=12)”. |
| Allocation concealment (selection bias) | Unclear risk | No information on allocation concealment. |
| Blinding of participants and personnel (performance bias)  All outcomes | Unclear risk | No information on the blinding of participants and personnel. |
| Blinding of outcome assessment (detection bias)  All outcomes | Unclear risk | No information on blindng of outcome assessment has been provieded. |
| Incomplete outcome data (attrition bias)  All outcomes | Low risk | No ITT (Intention to treat) analysis done |
| Selective reporting (reporting bias) | Low risk | All outcome variables reported in the results section |
| Other bias | Unclear risk | No other bias |

Characteristics of excluded studies [ordered by study ID]

| Study | Reason for exclusion |
| --- | --- |
| 2009 | Ineligible intervention |
| Abdollahpour 2018 | Ineligible Intervention |
| Abdul 2018 | Letter/Editorial |
| Abell 2007 | Letter/Editorial |
| Abell 2007a | Letter/Editorial |
| Abrams 2009 | Conceptual Paper |
| Adams 2006 | Conceptual Paper |
| Adis Medical Writers 2019 | Conceptual Paper |
| Ahmadi 2014 | Ineligible intervention |
| Ahmadpanah 2018 | Ineligible intervention |
| Ahokas 1998 | Ineligible intervention |
| Ahokas 1998a | Ineligible Intervention |
| Ahokas 1999 | Ineligible intervention |
| Ahokas 1999a | Ineligible intervention |
| Ahokas 2000 | Ineligible intervention |
| Ahokas 2000a | Ineligible intervention |
| Ahokas 2001 | Ineligible intervention |
| Ahokas 2001a | Ineligible Intervention |
| Akister 2004 | Ineligible intervention |
| Alberque 2008 | Conceptual paper |
| Albright 1993 | Conceptual Paper |
| Albright 2012 | Ineligible intervention |
| Alder 2002 | Ineligible intervention |
| Alexander 2013 | Ineligible intervention |
| Alhusen 2016 | Conceptual Paper |
| Ali 2010 | Ineligible intervention |
| Alley 1996 | Conceptual Paper |
| Altemus 2012 | Ineligible study design: descriptive non-experimental design |
| Altshuler 2001 | Expert Guidelines |
| Altshuler 2001a | Expert guidelines |
| Altshuler 2001b | Expert Guidelines |
| Altshuler 2002 | Ineligible intervention |
| Altın 2012 | Ineligible study design: Descriptive design |
| Alvarez 2015 | Conceptual Paper |
| Amidolare 1970 | Conceptual Paper |
| Amino 2002 | Conceptual Paper |
| Ammerman 2013 | Ineligible intervention |
| Ammerman 2013a | Ineligible intervention |
| Ammerman 2015 | Ineligible intervention |
| Anjum 2019 | Ineligible study design: Descriptive design |
| Anokye 2018 | Ineligible study design: Prevalence study design |
| Anton 2015 | Ineligible intervention |
| Appleby 1997 | Ineligible intervention |
| Arrais 2014 | Ineligible intervention |
| Ashford 2018 | Mothers other than LMICs |
| Bahk 2014 | Ineligible intervention |
| Bain 2014 | Mothers other than LMICs |
| Baker 2002 | Conceptual Paper |
| Baker-Ericzen 2008 | Ineligible intervention |
| Basu 2008 | Report |
| Basu 2008a | Authors’ Reply |
| Beeber 2007 | Mothers other than LMICs |
| Beeber 2013 | Mothers other than LMICs |
| Benhaïjoub 2008 | case study |
| Benmhammed 2019 | Ineligible intervention |
| Berle 2012 | Report |
| Bevan 2013 | Report |
| Bhat 2017 | Ineligible intervention |
| Bhatia 1999 | Conceptual paper |
| Birkhead 2013 | Ineligible intervention |
| Blackmore 2012 | Systematic review |
| Bland 2009 | Report |
| Bledsoe-Mansori 2013 | Mothers other than LMICs |
| Bloch 2011 | ineligible intervention |
| Bloch 2012 | Mothers other than LMICs |
| Blum 2003 | Letter/Editorial |
| Boath 1999 | Ineligible intervention |
| Brandon 2012 | Mothers other than LMICs |
| Brugha 2011 | Ineligible intervention |
| Buck 2019 | Report |
| Cauli 2019 | Conceptual Paper |
| Chabrol 2001 | Ineligible intervention |
| Chabrol 2002 | Ineligible intervention |
| Chun-Liu 2005 | Ineligible intervention |
| Chung 2015 | Case report |
| Clarici 2015 | Ineligible intervention |
| Clark 2008 | Mothers other than LMICs |
| Clark 2011 | Ineligible intervention |
| Cooper 2003 | Ineligible intervention |
| Danaher 2013 | Ineligible Intervention |
| Dennis 2004 | Systematic Review |
| Dennis 2007 | Systematic Review |
| Dennis 2009 | Ineligible intervention |
| Dennis 2009a | Ineligible intervention and population |
| Dennis 2012 | Mothers other than LMICs |
| Dennis 2013 | Systematic Review |
| Dennis 2014 | Systematic Review |
| Dennis 2015 | Mothers other than LMICs |
| Dennis 2020 | Mothers other than LMICs |
| Dere 2018 | Ineligible intervention |
| Di Venanzio 2017 | Conceptual Paper |
| Duffecy 2019 | Mothers other than LMICs |
| Dugravier 2013 | Mothers other than LMICs |
| Duval 2016 | Commentary |
| Duval 2016a | Commentary |
| Edmond 2017 | Letter |
| Epperson 1999 | Report |
| Fathi-Ashtiani 2015 | Ineligible intervention |
| Fathi-Ashtiani 2015a | Ineligible Intervention |
| Fitelson 2010 | Systematic Review |
| Flanagan 2011 | Ineligible intervention |
| Forman 2007 | Mothers other than LMICs |
| Ganga 2016 | Ineligible intervention |
| Gao 2010 | Ineligible intervention: Although the intervention is IPT-based Childbirth education but is not a complete IPT therapy. |
| Gao 2012 | Ineligible intervention: Although the intervention is IPT-based Childbirth education but is not a complete IPT therapy. |
| Genovez 2018 | Systematic Review |
| Gillibrand 2012 | Conceptual Paper |
| Gjerdingen 2003 | Ineligible Patient Population |
| Glangeaud-Freudenthal 2003 | Commentary |
| Glavin 2010 | Ineligible intervention |
| Glavin 2010a | Ineligible intervention |
| Goeser 2008 | Conceptual Paper |
| Goldbort 2005 | Commentary |
| Goldvarg 2011 | Mothers other than LMICs |
| Goodman 2008 | Mothers other than LMICs |
| Goodman 2013 | Ineligible setting |
| Goodman 2015 | Ineligible intervention |
| Grote 2003 | Conceptual Paper |
| Grote 2004 | Conceptual Paper |
| Grote 2004a | Ineligible population |
| Grote 2009 | Mothers other than LMICs |
| Grote 2011 | Mothers other than LMICs |
| Grote 2015 | Ineligible intervention |
| Grote 2016 | Ineligible intervention |
| Grote 2017 | Mothers other than LMICs |
| Gruen 1993 | Systematic Review |
| Guille 2017 | Mothers other than LMICs |
| Gureje 2019 | Mothers other than LMICs |
| Guscott 1991 | Conceptual Paper |
| Gutteridge 2002 | Systematic Review |
| Hall 2020 | Mothers other than LMICs |
| Hartl 2005 | Report |
| Hendrick 2003 | Report |
| Hendrick 2003a | Report |
| Hendrick 2003b | Conceptual Paper |
| Highet 2004 | Mothers other than LMICs |
| Hofecker-Fallahpour 2003 | Ineligible Intervention |
| Hofecker-Fallahpour 2003a | Ineligible Intervention |
| Hoffbrand 2001 | Systematic Review |
| Hohm 2017 | Ineligible study design: Descriptive design |
| Holden 1989 | Ineligible intervention |
| Homewood 2009 | Ineligible study design: Qualitative Study |
| Honey 2002 | Ineligible intervention |
| Horowitz 2006 | Ineligible study design: Descriptive study |
| Hou 2014 | Ineligible intervention |
| Howard 2004 | Systematic Review |
| Howard 2006 | Systematic Review |
| Howard 2015 | Report |
| Husain 2016 | Ineligible intervention |
| Husain 2016a | Ineligible Intervention |
| Husain 2017 | Ineligble intervention |
| Jermain 1995 | Conceptual Paper |
| Jiang 2014 | Ineligible intervention |
| Johnson 2016 | Mothers other than LMICs |
| Kamibeppu 2002 | Case study |
| Kao 2015 | Mothers other than LMICs |
| Kersten-Alvarez 2010 | Mothers other than LMICs |
| Kersting 2003 | Ineligible Intervention |
| Kettunen 2017 | Conceptual Paper |
| Klier 2001 | Mothers other than LMICs |
| Kopelman 2005 | Conceptual Paper |
| Kozinszky 2012 | Ineligible Outcomes |
| Kumar 2020 | Study Protocol with ineligible patient population |
| Kuosmanen 2010 | Mothers other than LMICs |
| KUOSMANEN 2010 | Mothers other than LMICs |
| Kurzweil 2008 | Ineligible intervention |
| Lara 2010 | Ineligible intervention |
| Lara 2013 | Ineligible study design: Descriptive study |
| Lau 2005 | Ineligible study design: Descriptive study |
| Le 2016 | PPD Prevention study |
| Lee 2016 | Conceptual Paper |
| Leis 2009 | Systematic Review |
| Lembke 2002 | Commentary |
| Lenze 2015 | Mothers other than LMICs |
| Lenze 2017 | Mothers other than LMICs |
| Lenze 2020 | Mothers other than LMICs |
| Letourneau 2011 | Ineligible intervention |
| Leung 2016 | ineligible intervention |
| Likierman 2003 | case study |
| Lin 2018 | Systematic Review |
| Lindensmith 2018 | Systematic Review |
| Ling-ling 2015 | Ineligible intervention |
| Loughnan 2018 | Ineligible intervention |
| Loughnan 2019 | Ineligible intervention |
| Lumley 2001 | Conceptual Paper |
| Lumley 2004 | Systematic Review |
| Lund 2014 | Ineligible intervention |
| Lund 2019 | Ineligible intervention |
| Luty 2013 | Ineligible intervention |
| Luty 2016 | Systematic Review |
| MacInnes 2000 | Ineligible intervention |
| Marrs 2013 | Ineligible intervention |
| McCarthy 1998 | Conceptual paper |
| McKay 2009 | Report |
| McKee 2006 | Mothers other than LMIC |
| McQueen 2008 | Guidelines |
| McVittie 2019 | Ineligible study design: Qualitative case stuy |
| Meager 1996 | Ineligible intervention |
| Mehri 2019 | Ineligible intervention |
| Meschino 2016 | Mothers other than LMICs |
| Milani 2015 | Ineligible intervention |
| Milgrom 2005 | Ineligible Intervention & Mothers other than LMICs |
| Milgrom 2011 | Ineligible intervention |
| Milgrom 2014 | Study protocol |
| Milgrom 2014a | Ineligible intervention |
| Milgrom 2015 | Ineligible intervention |
| Milgrom 2016 | Ineligible intervention |
| Miller 2008 | Mothers other than LMICs |
| Miniati 2014 | Systematic Review |
| Mirabella 2016 | Ineligible intervention |
| Miranda 2009 | Commentary |
| Misri 2006 | Mothers other than LMICs |
| Moayedoddin 2013 | Mothers other than LMICs |
| Moel 2010 | Mothers other than LMICs |
| Morrell 2009 | Mothers other than LMICs |
| Morrell 2009a | Ineligible Population |
| Morris 1987 | Ineligible Intervention |
| Morris 1987a | Ineligible Intervention |
| Moss 2009 | Conceptual Paper |
| Mulcahy 2010 | Mothers other than LMICs |
| Mulcahy 2010a | Mothers other than LMICs |
| Murray 2003 | Mothers other than LMICs |
| Muzik 2009 | Report |
| Nanzer 2012 | Mothers other than LMICs |
| Nasreen 2013 | Ineligible study design: descriptive study |
| Naysmith 2015 | Mothers other than LMICs |
| NCT00043602 2002 | Study Protocol & mothers other than LMICs |
| NCT00051246 2003 | Study Protocol & mothers other than LMICs |
| NCT00053651 2003 | Ineligible intervention |
| NCT00251342 2005 | Study protocol & Ineligible intervention |
| NCT00436150 2007 | Study Protocol & Mothers other than LMICs |
| NCT00602355 2008 | Mothers other than LMICs |
| NCT01028482 2009 | Ineligible Intervention |
| NCT01482832 2011 | Mothers other than LMICs |
| NCT02057627 2014 | Study Protocol |
| NCT02191423 2014 | Ineligible intervention |
| NCT03430622 2018 | On-going study in other than LMIC |
| NCT03499756 2018 | On-going study in other than LMIC |
| NCT04138368 2019 | Ineligible intervention |
| Noe 2018 | Report |
| Norris 2013 | Mothers other than LMICs |
| Nylen 2010 | Mothers other than LMICs |
| O’Hara 2000 | Mothers other than LMICs |
| O’Hara 2019 | Mothers other than LMICs |
| O’Mahen 2013 | Mothers other than LMICs |
| O’Mahen 2014 | Ineligible intervention |
| Oddo 2008 | Ineligible outcome variables |
| Oreizi 2017 | Systematic Review |
| Paris 2009 | Mothers other than LMICs |
| Paris 2011 | Mothers other than LMICs |
| Pellet 2014 | Ineligible patient population |
| Pessagno 2013 | Mothers other than LMICs |
| Phipps 2013 | Mothers other than LMICs |
| Pinheiro 2008 | Case studies |
| Pinheiro 2014 | Ineligible intervention |
| Poobalan 2007 | Systematic review |
| Posmontier 2013 | Mothers other than LMICs |
| Posmontier 2015 | Mothers other than LMICs |
| Posmontier 2015a | Mothers other than LMICs |
| Posmontier 2016 | Mothers other than LMICs |
| Posmontier 2019 | Mothers other than LMICs |
| Puckering 2010 | Mothers other than LMICs |
| Rabiepoor 2019 | Conceptual Paper |
| Rahioui 2015 | Conceptual Paper |
| Rahman 2007 | Ineligible study design: Qualitative Study |
| Rahman 2013 | Systematic Review |
| Reay 2002 | Report |
| Reay 2005 | Mothers other than LMICs |
| Reay 2006 | Mothers other than LMICs |
| Reay 2006a | Erratum |
| Reay 2006b | Mothers other than LMICs |
| Reay 2012 | Commentary |
| Reck 2004 | Conceptual Paper |
| Reck 2004a | Commentary |
| Reck 2007 | Conceptual Paper |
| Rojas 2007 | Ineligible intervention |
| Rojas 2007a | Ineligible Intervention |
| Rosenberg 2009 | Ineligible intervention |
| Sadeghi 2017 | Conceptual Paper |
| Sampson 2016 | Ineligible intervention |
| Sanford 2002 | Study Protocol & Mothers other than LMICs |
| Schneider 2008 | Ineligible patient population |
| Scott 2008 | Commentary |
| Seeman 2001 | Mothers other than LMICs |
| Segre 2004 | Commentary |
| Segre 2011 | Report |
| Seneviratne 2011 | Commentary |
| Shanok 2007 | Qualitative study & Mothers other than LMICs |
| Shapiro 2013 | Systematic Review |
| Sharp 2010 | Mothers other than LMICs |
| Shulman 2018 | Ineligible intervention |
| Singla 2017 | Systematic Review |
| Smith 2012 | Conceptual Paper |
| Sockol 2018 | Systematic Review |
| Song 2015 | Systematic Review |
| Spinelli 2003 | Mothers other than LMICs |
| Spinelli 2013 | Mothers other than LMICs |
| Spinelli 2016 | Mothers other than LMICs |
| Stamou 2018 | Systematic Review |
| Stephens 2016 | Systematic Review |
| Stuart 1995 | Conceptual Paper |
| Stuart 1995a | Letter |
| Stuart 2001 | Conceptual Paper |
| Stuart 2003 | Systematic Review |
| Stuart 2008 | Conceptual Paper |
| Stuart 2008a | Mothers other than LMICs |
| Stuart 2012 | Commentary |
| Stuart 2014 | Systematic Review |
| Suri 2001 | Ineligible intervention |
| Susman 1996 | Conceptual Paper |
| Swartz 2014 | Systematic Review |
| Swartz 2018 | Ineligible patient population |
| Sword 2005 | Systematic Review |
| Tait 2010 | Ineligible study design: Descriptive Study |
| Tambelli 2015 | Mothers other than LMICs |
| Tareen 2013 | Mothers other than LMICs |
| Toru 2018 | Ineligible study design: Descriptive Study |
| Toth 2013 | Mothers other than LMICs |
| Truitt 2013 | Ineligible intervention |
| Tsivos 2015 | Ineligible intervention |
| Turella 2011 | Ineligible study design: Review paper |
| Van 2018 | Ineligible intervention |
| Vasa 2014 | Ineligible study design: Descriptive Study |
| Vogel 2011 | Report |
| Vythilingum 2013 | Ineligible study design: Descriptive Study |
| Webster 2011 | Ineligible intervention |
| Weinberg 2013 | Ineligible study design: Descriptive study |
| Weissman 2007 | Commentary |
| Wickberg 1996 | Ineligible intervention |
| Wickberg 2001 | Wrong setting |
| Wisner 2002 | Report |
| Wisner 2017 | Mothers other than LMICs |
| Wozney 2017 | Ineligible intervention |
| Wu 2008 | Ineligible intervention |
| Yang 2019 | Mothers other than LMICs |
| Yator 2020 | Study Protocol of the included study |
| Yawn 2012 | Ineligible intervention |
| Zafar 2016 | Inelgible Intervention |
| Zayas 2008 | Mothers other than LMICs |
| Zlotnick 2001 | Report |

Appendices

Appendix 1. Authors ‘ d eclaration

Authors’ responsibilities

By completing this form, you accept responsibility for preparing, maintaining and updating the review in accordance with Campbell Collaboration policy. Campbell will provide as much support as possible to assist with the preparation of the review.

A draft review must be submitted to the relevant Coordinating Group within two years of protocol publication. If drafts are not submitted before the agreed deadlines, or if we are unable to contact you for an extended period, the relevant Coordinating Group has the right to de-register the title or transfer the title to alternative authors. The Coordinating Group also has the right to de-register or transfer the title if it does not meet the standards of the Coordinating Group and/or Campbell.

You accept responsibility for maintaining the review in light of new evidence, comments and criticisms, and other developments, and updating the review at least once every five years, or, if requested, transferring responsibility for maintaining the review to others as agreed with the Coordinating Group.

Publication in the Campbell Library

The support of the Coordinating Group in preparing your review is conditional upon your agreement to publish the protocol, finished review, and subsequent updates in the Campbell Library. Campbell places no restrictions on publication of the findings of a Campbell systematic review in a more abbreviated form as a journal article either before or after the publication of the monograph version in Campbell Systematic Reviews. Some journals, however, have restrictions that preclude publication of findings that have been, or will be, reported elsewhere and authors considering publication in such a journal should be aware of possible conflict with publication of the monograph version in Campbell Systematic Reviews. Publication in a journal after publication or in press status in Campbell Systematic Reviews should acknowledge the Campbell version and include a citation to it. Note that systematic reviews published in Campbell Systematic Reviews and co-registered with Cochrane may have additional requirements or restrictions for co-publication. Review authors accept responsibility for meeting any co-publication requirements.

**I understand the commitment required to undertake a Campbell review, and agree to publish in the Campbell Library. Signed on behalf of the authors**:

| **Form completed by: Harmeet Kaur Kang** | **Date: January 2nd, 2024** |
| --- | --- |

Appendix 2. Search Results

| **Search Results** | | | | | | |
| --- | --- | --- | --- | --- | --- | --- |
| **Database** | **Search terms** | **Date of search** | **Number of hits** | **Notes** | **Second Search** | **New results** |
| CINAHL | (“postpartum depressi*” OR “post-partum depressi*” OR “postnatal depressi*” OR “post-natal depressi*” OR ppd OR pnd OR (MH “Depression, Postpartum”))  AND  (psychotherap* OR therap* OR counsel* OR (MH “Psychotherapy”)) | 27.01.2020 | 1,929 |  | 08.07.2022 | 427 |
| PsycINFO | (“postpartum depressi*” OR “post-partum depressi*” OR “postnatal depressi*” OR “post-natal depressi*” OR ppd OR pnd OR (DE “Postpartum Depression”))  AND  (psychotherap* OR therap* OR counsel* OR (DE “Interpersonal Psychotherapy”)) | 31.01.2020 | 1,607 |  | 20.07.2022 | 307 |
| PubMed | (“postpartum depressi*” OR “post-partum depressi*” OR “postnatal depressi*” OR “post-natal depressi*” OR ppd OR pnd OR (“depression, postpartum”[MeSH Terms]))  AND  (psychotherap* OR therap* OR counsel* OR (“psychotherapy”[MeSH Terms])) | 10.02.2020 | 5,027 |  | 18.07.2022 | 1,406 |
| Web of Science | (“postpartum depressi*” OR “post-partum depressi*” OR “postnatal depressi*” OR “post-natal depressi*” OR ppd OR pnd)  AND  (psychotherap* OR therap* OR counsel*) | 10.02.2020 | 2,308 | No Thesaurus terms available in this database. | 18.07.2022 | 981 |
| Cochrane | (“postpartum depressi*” OR “post-partum depressi*” OR “postnatal depressi*” OR “post-natal depressi*” OR ppd OR pnd OR (MeSH descriptor: [Depression, Postpartum]))  AND  (psychotherap* OR therap* OR counsel* OR [MeSH descriptor: [Psychotherapy])) | 11.02.2020 | 1,061 trials, 43 reviews |  | 12.07.2022 | 717 |
| ERIC (via Ebsco) | (“postpartum depressi*” OR “post-partum depressi*” OR “postnatal depressi*” OR “post-natal depressi*” OR ppd OR pnd)  AND  (psychotherap* OR therap* OR counsel* OR (DE “Psychotherapy”)) | 14.02.2020 | 27 | There was no Thesaurus term for postpartum depression in this database | 08.07.2022 | 0 |
| Embase (via HDA) | (‘postnatal depression’/ OR ‘postpartum depressi*‘ OR ‘post-partum depressi*‘ OR ‘postnatal depressi*‘ OR ‘post-natal depressi*‘ OR ppd OR pnd) AND (‘psychotherapy’/ OR psychotherap* OR therap* OR counsel* | 03.04.2020 | 3,767 |  |  |  |
|  |  |  |  |  |  | 3,812 |
